# Typing myalgic encephalomyelitis by infection at onset: A DecodeME study

**DOI:** 10.3310/nihropenres.13421.1

**Published:** 2023-04-24

**Authors:** Andrew D. Bretherick, Simon J. McGrath, Andy Devereux-Cooke, Sian Leary, Emma Northwood, Anna Redshaw, Pippa Stacey, Claire Tripp, Jim Wilson, Sonya Chowdhury, Isabel Lewis, Øyvind Almelid, Sumy V. Baby, Tom Baker, Hannes Becher, Thibaud Boutin, Malgorzata Clyde, Diana Garcia, John Ireland, Shona M. Kerr, Ewan McDowall, David Perry, Gemma L. Samms, Veronique Vitart, Jareth C. Wolfe, Chris P. Ponting

**Affiliations:** 1 MRC Human Genetics Unit, The University of Edinburgh, Edinburgh, Scotland, EH4 2XU, UK; 2 Pain Service, Ninewells Hospital and Medical School, NHS Tayside, Dundee, Scotland, DD1 9SY, UK; 3 c/o DecodeME, MRC Human Genetics Unit, The University of Edinburgh, Edinburgh, Scotland, EH4 2XU, UK; 4 42 Temple Street, Keynsham, Action For ME, Bristol, England, BS31 1EH, UK

**Keywords:** Myalgic encephalomyelitis, Post-viral syndrome, Postexertional malaise, Sex-bias, Sub-types

## Abstract

**Background:**

People with myalgic encephalomyelitis / chronic fatigue syndrome (ME/CFS) daily experience core symptoms of post-exertional malaise, unrefreshing sleep, and cognitive impairment or brain fog. Despite numbering 0.2-0.4% of the population, no laboratory test is available for their diagnosis, no effective therapy exists for their treatment, and no scientific breakthrough regarding their pathogenesis has been made. It remains unknown, despite decades of small-scale studies, whether individuals experience different types of ME/CFS separated by onset-type, sex or age.

**Methods:**

DecodeME is a large population-based study of ME/CFS that recruited 17,074 participants in the first 3 months following full launch. Their detailed questionnaire responses provided an unparalleled opportunity to investigate illness severity, onset, course and duration.

**Results:**

The well-established sex-bias among ME/CFS patients is evident in the initial DecodeME cohort: 83.5% of participants were females. What was not known previously was that females’ comorbidities and symptoms tend to be more numerous than males’. Moreover, being female, being older and being over 10 years from ME/CFS onset are significantly associated with greater severity. Five different ME/CFS onset types were examined in the self-reported data: those with ME/CFS onset (i) after glandular fever (infectious mononucleosis); (ii) after COVID-19 infection; (iii) after other infections; (iv) without an identified infectious onset; and, (v) where the occurrence of an infection at or preceding onset is not known.

**Conclusions:**

This revealed that people with a ME/CFS diagnosis are not a homogeneous group, as clear differences exist in symptomatology and comorbidity.

## Plain English summary

Myalgic Encephalomyelitis / Chronic Fatigue Syndrome (ME/CFS) is a chronic disease affecting an estimated 250,000 people in the UK. Its defining symptom is post-exertional malaise, the disproportionate worsening of symptoms following even minor physical or mental exertion. For many people, ME/CFS means a substantial impairment of their activity levels, a high level of disability and a poor quality of life. Research questions recently prioritised by people with ME/CFS and doctors included: “Are there different types of ME/CFS linked to different causes and how severe it becomes? Do different types of ME/CFS need different treatments or have different chances of recovery?” These can begin to be addressed from the questionnaire responses given by more than 17 thousand people with ME/CFS to the DecodeME study.

Results show that people with ME/CFS do not form a single uniform group, because they record clear differences in symptoms and co-occurring medical conditions. For example, if someone reported an infection just prior to getting ME/CFS, then this affects the chance that they report specific symptoms or co-occurring conditions.

It is well known that most people with ME/CFS are females. What was not clear previously was that females tend to have more symptoms and more co-occurring conditions. Also, being female, being older and being over 10 years from ME/CFS onset are associated with greater severity of ME/CFS symptoms.

These findings could indicate that by studying each ME/CFS type separately – rather than analysing all ME/CFS patients together – it will be easier to understand what has gone wrong in the illness.

## Introduction

Myalgic encephalomyelitis / chronic fatigue syndrome (ME/CFS) is a chronic multisystem disorder that affects an estimated 0.2–0.4% of the UK population ^1, 2^. Its core symptoms are post-exertional malaise, pain, fatigue, unrefreshing sleep, cognitive impairment and/or orthostatic intolerance that may change across the life-course ^3^. Many people with ME/CFS report an infectious episode prior to their initial symptoms. Up to 10% of people with glandular fever (also known as infectious mononucleosis) are eventually diagnosed with ME/CFS ^4, 5^, with similar fractions of people with Ross River virus or *Coxiella burnetii* infections also developing ME/CFS ^4^. Long COVID, whose symptoms can overlap those of ME/CFS, appears to arise at a similar rate after infection with severe acute respiratory syndrome coronavirus-2 (SARS-CoV-2) ^6, 7^. Onset of ME/CFS can also occur without report of infection ^8^. Pathogenesis is unknown, and effective treatment is not available. In one study, the health-related quality of life for people with ME/CFS was worse than 20 other conditions compared, including breast, prostate, colon or lung cancer, type I or II diabetes, stroke, multiple sclerosis and schizophrenia ^9^.

One priority from a 2022 priority setting exercise facilitated by the James Lind Alliance ^10^ was “Are there different types of ME/CFS linked to different causes and how severe it becomes? Do different types of ME/CFS need different treatments or have different chances of recovery?” To address this question, we took advantage of questionnaire data from DecodeME, a new study launched in the UK in September 2022. Before the end of the year, over 17,000 people with a ME/CFS diagnosis from a health professional, and at least 16 years (y) old, had been recruited and completed the study questionnaire.

Over many decades, ME/CFS studies have addressed similar questions using symptom data for tens or hundreds of participants recruited using various inclusion and exclusion criteria ^8, 11, 12^. However, they remain inconclusive on whether different ME/CFS types exist and whether symptoms are sex-biased. The DecodeME project provided a unique opportunity to perform adequately-powered analyses for detecting differences within a single large ME/CFS cohort, under an assumption that ME/CFS type is delineated by onset type.

## Methods

### Patient and Public Involvement

The DecodeME project grew out of the UK ME Research Collaborative (MERC), formerly known as the CFS/M.E. Research Collaborative or CMRC, which was first established in 2013. The MERC includes people with ME/CFS and carers within a Patient Advisory Group (PAG). As the project evolved in 2018–19, PPI was embedded in every discussion and workshop, resulting in the project becoming a co-production with its grant proposal, aims and outcomes being decided by researchers and PPI in equal measure. In 2020, PPI Steering Group members were selected from across diverse charities and organisations, and for their breadth of experience. In DecodeME, PPI representatives serve on each of its delivery groups, lead on marketing and communication (including social media), and contribute the majority (two of three) members of the decision-making body, the Management Group. People with lived experience of ME/CFS led the co-creation of a new DecodeME questionnaire, making substantial improvements in comprehension and accuracy, thereby boosting recruitment. The project’s name was suggested and decided by PPI members.

DecodeME’s genetics question (“What, if any, significant genetic differences are there between people with—and those without—ME/CFS?") was identified as a priority first by the MERC and its PAG, before being confirmed as a priority by a wider section of the patient community in the results of the Priority Setting Partnership for ME/CFS ^10^. Established participant selection criteria were further refined with PPI throughout. PPI members, through their profound understanding of ME/CFS phenotypes, triggers, severity, symptom range, comorbidities and more, have improved the study’s adherence to our chosen case definition and thus further assured the relevance of genetic associations to ME/CFS lived experience.

A substantial minority of volunteer participants who trialled an initial questionnaire reported difficulties when answering its questions. We then created a new version implementing Canadian Consensus and IOM/NAM criteria ^3, 13^ as well as criteria introduced in response to peer reviewers’ comments. This DecodeME questionnaire is freely available from the DecodeME website. As a co-production, PPI members advised and helped to create both our recruitment strategy and recruitment materials. Further description of DecodeME’s recruitment methods and PPI aspects can be found elsewhere ^14^. Before study launch public awareness of DecodeME was enhanced using regular podcasts, webinars, blog posts and media interviews. These media channels will be used by PPI members and scientists to disseminate results to the international ME/CFS community. PPI team members maintain extensive input into reporting of the results of the questionnaire (including in this article), providing greater understanding and context, and ensuring accessibility. Our GWAS plan was co-created by researchers and PPI members.

The DecodeME study was reviewed and given a favourable opinion by the North West – Liverpool Central Research Ethics Committee (21/NW/0169). Potential participation bias due to internet use was mitigated by providing a paper questionnaire and providing participants with assistance in completing their online questionnaires. Team members were available to answer phone calls and emails during working hours.

### Cohort

Participants were asked for their sex assigned at birth and about their conditions: “If a health professional has ever told you that you had any of the conditions below, please select all that apply. If the conditions don’t apply to you, please do not select any box.” Participants indicated whether each condition was Active (“If the condition has given you symptoms in the past 6 months”) or not active “If the condition has not given you symptoms in the past 6 months, either because it has died down or treatment has controlled it”). They were also asked about their symptoms: “In the last 6 months, have you had any of the symptoms below often, repeatedly, or constantly? Please mark any that apply. If none apply, leave all the boxes blank.” Questionnaire responses from participants who both consented to participate and self-reported being given a diagnosis of ME, CFS, ME/CFS or CFS/ME by a health professional (as of 19 December 2022) were analysed. Only those whose sex assigned at birth was male or female were analysed due to insufficient numbers of other identities. Participant ages were as of 19 December 2022. Further analyses will be undertaken for the full DecodeME cohort once the recruitment phase of the project is completed.

***Significance testing.*** Logistic regression analysis for Figure 6, for example, was of the form: OnsetType ~ age + sex + symptoms + intercept. The analyses conducted were: (i) for 80 symptoms against age and sex – Figure 4; (ii) for each symptom (n=80; and, age and sex) against severity – Figure 5; (iii) for each of the 5 onset types against 8 fatigue symptoms plus age and sex – Figure 6A; (iv) for each of the 5 onset types against 72 non-fatigue symptoms (plus age and sex) – Figure 6B; (v) for each of the 5 onset types against 5 illness courses, relative to ‘Fluctuating’, the majority response – Figure 6C; and (vi) for each of the 5 onset types against 34 comorbidities (active and inactive) plus age and sex – Figure 7. Analyses used the glm function in R version 4.2.2. Only *p*-values that survive Bonferroni correction for multiple tests per analysis are shown.

## Results

**Figure 1.**
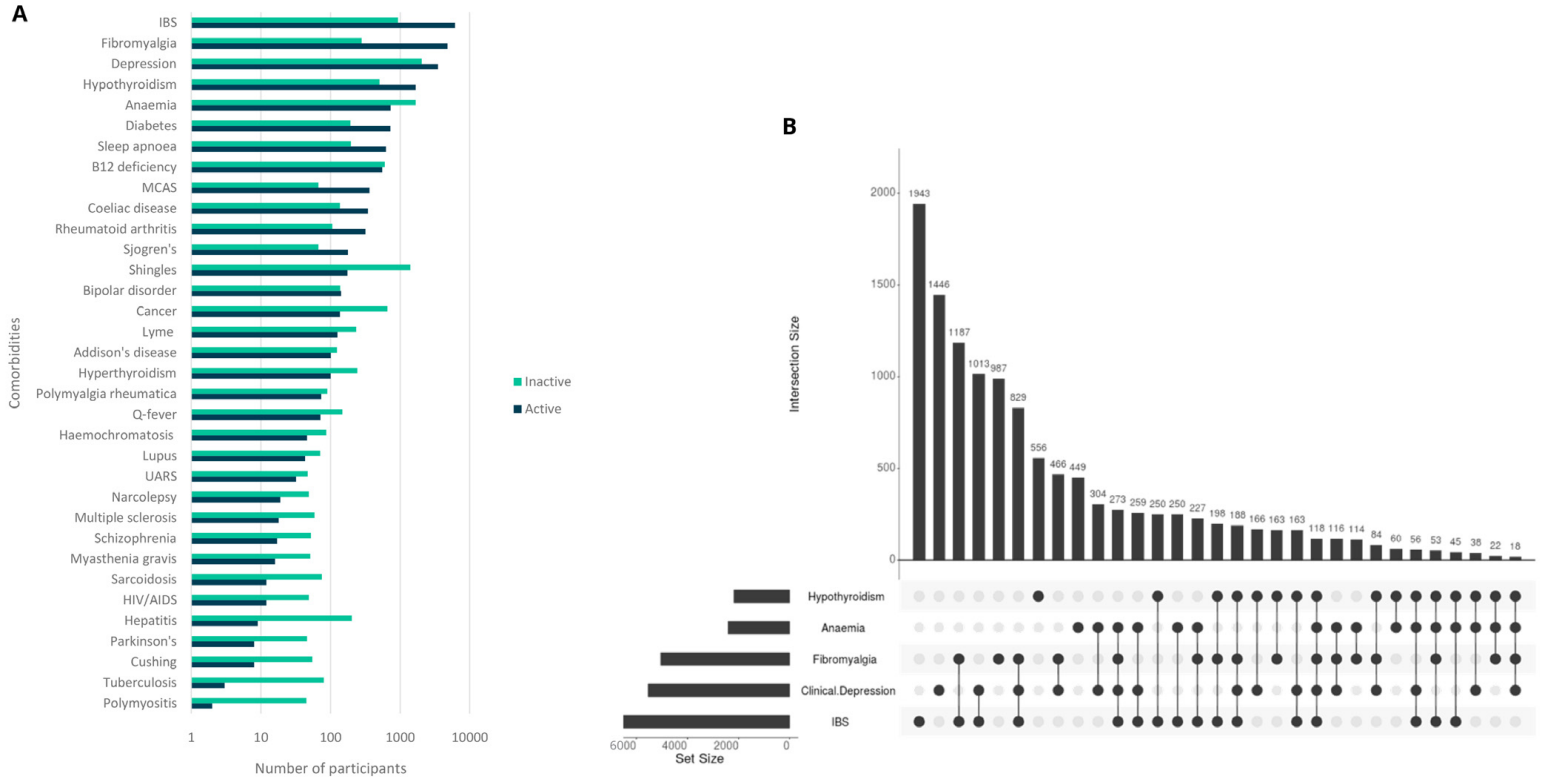
Numbers of DecodeME participants reporting conditions co-occurring with ME/CFS (comorbidities); total, 17074 participants. In ( **A**) numbers are shown in log _10_-scale and those with active or inactive comorbidities are indicated in blue or green, respectively. The UpSet plot ^17^ ( **B**) shows numbers of participants with five conditions that most frequently co-occur with ME/CFS. These either co-occur together with others (indicated by filled circles linked by lines) or else separately (filled circles not linked by lines).

**Figure 2.**
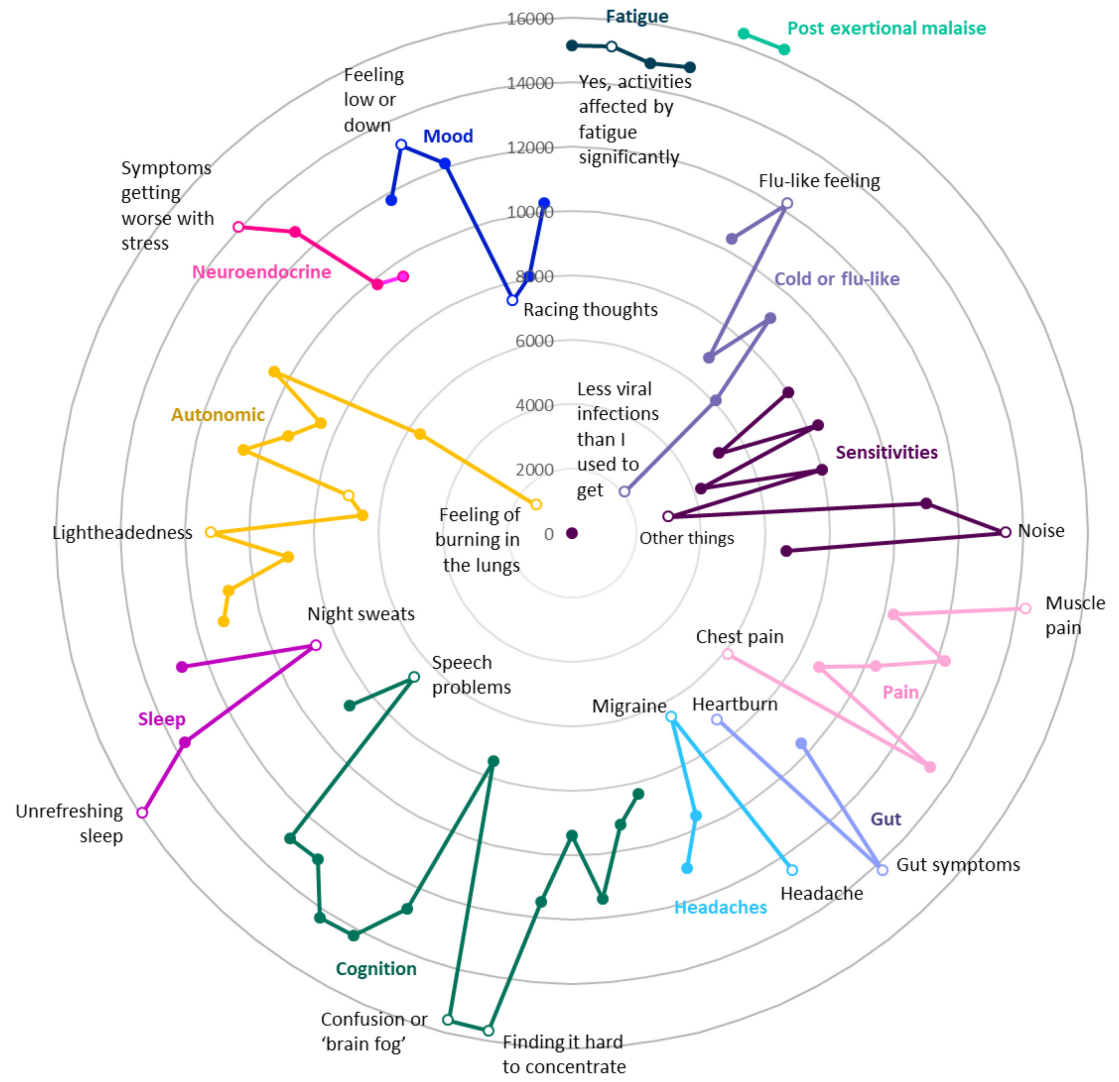
Numbers of DecodeME participants reporting symptoms (Radar chart); total, 17074 participants. Most frequently reported symptoms are furthest from these circles’ centre. Twelve different groups of questions are indicated in separate colours; for each symptom group, the most and least frequently reported symptoms are listed and indicated as unfilled circles. With reference to the DecodeME questionnaire ( www.decodeme.org.uk/app/uploads/2022/08/DecodeME-Questionnaire.pdf) the questions (Q) are, clockwise: Fatigue (Q8-answer 3 [Q8-3], Q9-3, Q3-1, Q10-1), Postexertional malaise (PEM, Q12-1 AND Q13-1), Cold or flu-like (Q14-4, -2, -1, -5, -6, -3), Sensitivities (Q15-1, -2, -3, -5, -7, -9, -4, -6, -8), Pain (Q16-4, -6, -5, -3, -2, -7, -1), Gut (Q17-1, -2, -3), Headaches (Q18-1, -4, -2, -3), Cognition (Q19-15, -7, -8, -9, -12, -3, -1, -2, -6, -10, -5, -13, -4, -14, -11), Sleep (Q20-4, -3, -2, -1), Autonomic (Q21-3, -6, -11, -10, -9, -4, -2, -1, -5, -7, -12, -8), Neuroendocrine (Q22-3, -1, -2, -4), and Mood (Q23-2, -3, -1, -5, -6, -4).

Between its full launch date of September 12, 2022 and a data freeze performed on December 19, 2022, DecodeME recruited 17,074 female or male participants who completed a questionnaire either online (98.1%) or with a paper version (1.9%) and consented to take part. Participants reported being diagnosed with ME/CFS by a health professional and were asked how long they have had their illness. Participants’ information included how long they had experienced ME/CFS symptoms, as well as whether they have any of 34 comorbidities (co-occurring conditions; Figure 1) or 82 symptoms (9 fatigue- and 73 non-fatigue symptoms; Figure 2). For each comorbidity, participants could indicate whether this was ‘active’ or ‘inactive’, meaning whether or not symptoms had been experienced in the preceding 6 months ( Figure 1A).

50.6% of participants reported two or more conditions co-occurring with ME/CFS, most commonly irritable bowel syndrome (IBS; 41.3%), clinical depression (32.4%) and fibromyalgia (29.5%), anaemia (14.1%) and hypothyroidism (12.8%). These results are similar to those of a previous study ^15^. Fibromyalgia and IBS occur together with ME/CFS for 18.0% of participants ( Figure 1B). 22.6% report no comorbidities.

DecodeME participants’ most frequent symptom is post-exertional malaise, a cardinal symptom of ME/CFS ^3^, followed by unrefreshing sleep, brain fog, fatigue, muscle pain and gut symptoms ( Figure 2). Almost all answered that once they had exceeded their energy limit their change in symptoms lasts “a long time, which can be more than 24 hours” (97.6%) and agreed that their fatigue affected them both physically and mentally (96.2%). For 88.7%, their fatigue occurs more than half of the time and 87.3% report their fatigue as disabling. Most participants (58.0%) indicated that their ME/CFS is “Fluctuating (my symptoms vary day to day but don’t go away)”, 12.7% describe their symptoms as “Relapsing and remitting (good periods with no symptoms alternating with symptomatically bad periods)” and 15.3% indicate their symptoms are “Getting worse”, similar to previous research ^15^.

**Figure 3.**
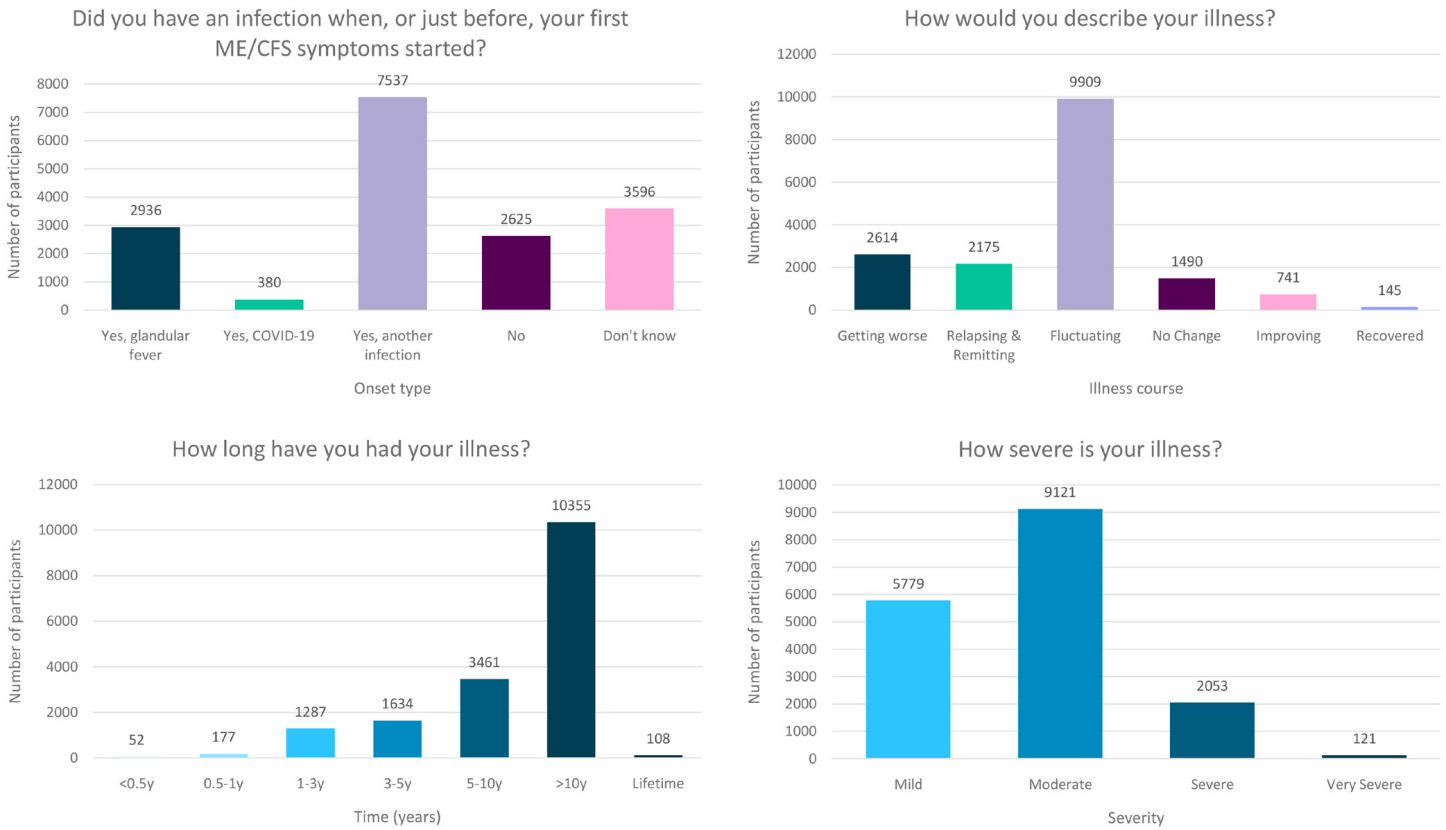
Onset type, illness course, duration of illness and severity of the DecodeME cohort. Numbers of DecodeME participants reporting whether they had an infection prior to ME/CFS onset (top left) as well as their illness’ course (top right), duration (bottom left) and severity (bottom right; *n* = 17,074 participants).

Participants were asked: “Did you have an infection when, or just before, your first ME/CFS symptoms started?” with five possible responses: (i) Yes, glandular fever ( *n*=2,936), (ii) Yes, COVID-19 ( *n*=380), (iii) Yes, another infection ( *n*=7,537), (iv) No ( *n*=2,625), or (v) Don’t know ( *n*=3,596; Figure 3). Proportions of DecodeME participants reporting glandular fever or another infectious disease prior to onset (17.2% or 63.5%, respectively) are similar to those previously reported ^11, 15, 16^. Proportions of people in the first 3 categories reporting a positive laboratory test of their infection prior to ME/CFS were 68.4%, 50.5% and 25.9%, respectively.

### Demographics

The DecodeME study continues to recruit individuals living in the UK aged 16y and above, with its oldest participant aged over 90y old. Male participants tended to be older than females (median 52y and 48y respectively; p < 2.2×10 ^-16^, Wilcoxon rank sum test). The median age of study participants was 49y, similar to those in previous USA-based studies ^18– 20^. Only 3.3% of 17,074 participants did not self-report their ethnicity as White, far fewer than the 18.3% in England and Wales who identify as non-White ( https://www.ethnicity-facts-figures.service.gov.uk/). Participants indicated the duration of their ME/CFS illness by selecting from a set of predefined ranges, for example between 5 and 10 years, or over 10 years, since onset of symptoms. Most (61.3%) DecodeME participants have had ME/CFS for over 10y, and 81.5% over 5y ( Figure 3). Together, study participants have experienced over 1.3 ×10 ^5^ years of ME/CFS symptoms.

Participants who started their illness within the last 1–3y or 0.5–1y numbered 1,287 or 354, respectively. These numbers are 57% and 21% fewer, per year, than the study’s 1,634 participants from the 3–5y recruitment interval. This paucity of participants with illness duration within 3 years may reflect how long it usually takes to receive a clinical diagnosis in the UK ( median 13–24 months). 

To examine the incidence of ME/CFS, we considered 3,150 participants reporting ME/CFS onset within the last 5y. Their median age was 40y, overlapping the peak age (40–59y) of ME/CFS diagnosis in UK primary care ^21^. On average, those with glandular fever onset <5y ago were a decade younger than all others (median ages 30.5y and 41y, respectively; *p* < 2.2×10 ^-16^, Wilcoxon rank sum test). This means that for at least half of our glandular fever onset participants, ME/CFS onset occurred after the age of 25y. This is a decade after peak incidence of glandular fever in the UK between 15 and 19y old ^22^. This difference in peak incidence is consistent with adolescents being less likely, than older people, to develop ME/CFS after glandular fever. 

### Sex- and age-bias of ME/CFS comorbidities and symptoms

In the DecodeME cohort, females outnumber males by over five-to-one (83.5% females; 16.5% males). This is among the highest female-bias among those with ME/CFS yet reported internationally ^3, 9, 21, 23– 27^. Despite this strong bias, the substantial number of males participating in DecodeME ( *N*=2,827) allowed the study to reveal previously unreported sex-biases in comorbidities or symptoms. 

Females with ME/CFS reported more comorbidities and symptoms than males in the DecodeME questionnaire. Two-thirds (66.7%) of females, but a half (52.7%) of males, reported at least one active comorbidity; similarly 39.2% of females and 28.6% of males reported at least one inactive comorbidity. Female participants reported, on average, more symptoms than males (42 versus 36).

**Figure 4.**
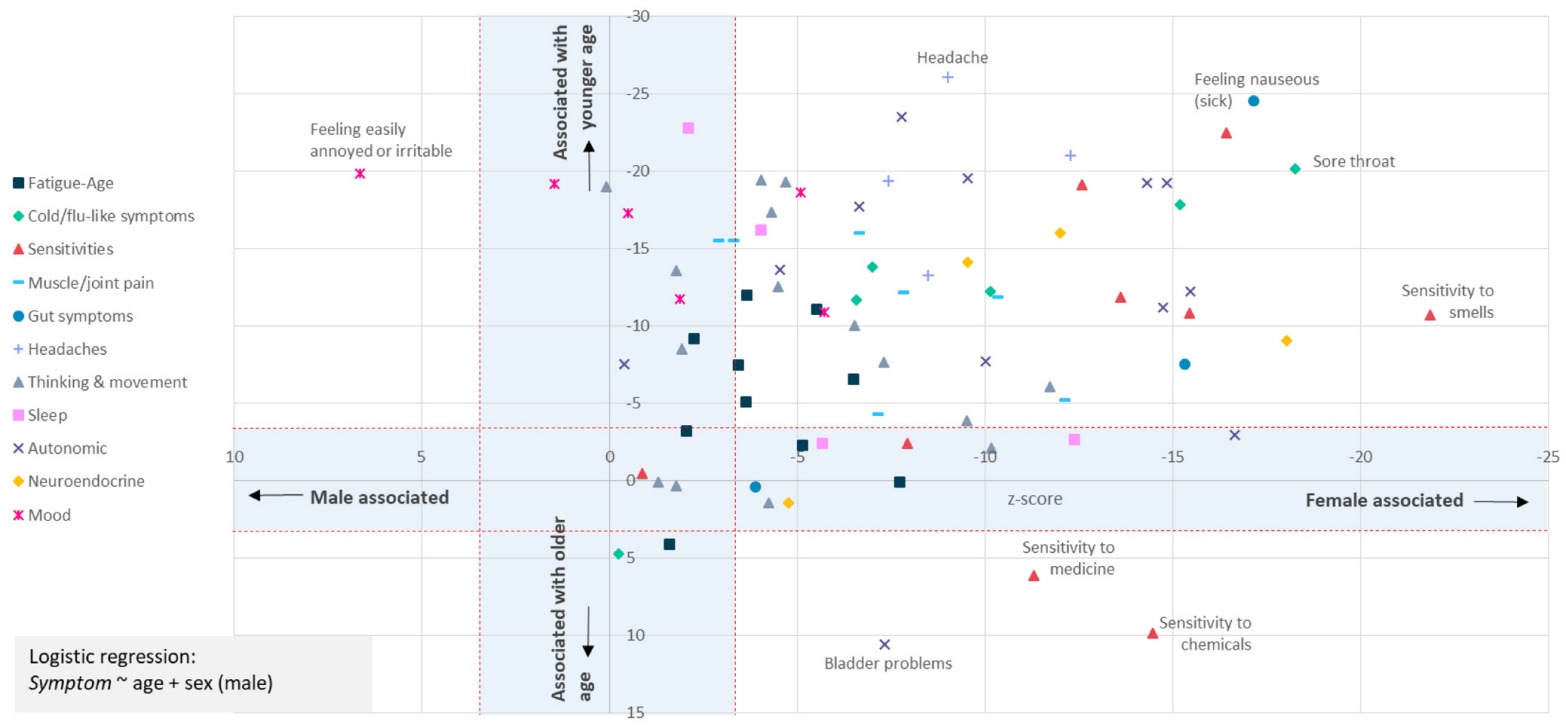
Most symptoms are strongly associated with female sex at birth and younger age. The question asked was: “In the last 6 months, have you had any of the symptoms below often, repeatedly, or constantly? Please mark any that apply.” Sex-biased (X-axis) and/or Age-biased (Y-axis) associations in a logistic regression analysis ( *Symptom* ~ age + sex + intercept) are shown as data points. Data points within the blue-shaded areas are not significant after accounting for 82 tests ( *p*<0.05/82, or |Z|<3.427. Only one symptom (“Feeling easily annoyed or irritable”) was male-biased; 3 symptoms (sensitivities to chemicals or medicine, or bladder problems) were associated with older age. Results for 80 symptoms are shown.

To test more formally for an association between age and sex and each symptom we used logistic regression using the Bonferroni correction to adjust for multiple tests ( Methods). This identified 62 of 80 symptoms as significantly female-biased, and 61 as biased towards younger age ( Figure 4). Female-bias is evident across all symptom types ( Figure 4). Females were significantly more likely to report fatigue ‘often, repeatedly, or all the time’ ( *p=*6×10 ^-4^; age *p*=1.1×10 ^-13^), and more likely to report post-exertional malaise after physical or mental activity ( *p*=3×10 ^-4^; age *p*=4.2×10 ^-7^).

### Severity, comorbidities and symptoms

Participants were asked: “How severe is your illness?” with answer options matching severity definitions from the UK’s National Institute for Health and Care Excellence (NICE) guidelines (2021). Most DecodeME participants’ severity levels are categorised as Mild or Moderate, but Severe and Very Severe individuals are also represented ( Figure 3). Severity categories were consistent with participants’ reports of their comorbidities and symptoms (see below).

Being female, increasing age and being over 10y from ME/CFS onset are each separately associated with severity in the DecodeME cohort (sex: *p*=4.5×10 ^-4^; age: *p*<2.2×10 ^-16^; years since ME/CFS onset: *p*=1.6×10 ^-6^). These results are from a comparison of those with mild ME/CFS (34%) against the remaining 66% with moderate, severe or very severe illness. Testing for all 68 co-occurring (active and inactive) comorbidities, and including both age and sex as covariates in the model, 6 active comorbidities were significantly associated with severity. In order of decreasing significance these were: fibromyalgia ( *p*<2×10 ^-16^), clinical depression ( *p*<2×10 ^-16^), irritable bowel syndrome ( *p*=5.7×10 ^-12^), mast cell activation syndrome ( *p*=1.8×10 ^-11^), diabetes ( *p*=9.5×10 ^-10^) and sleep apnoea ( *p*=5.2×10 ^-8^). Severity was also associated with a single inactive comorbidity, hypothyroidism ( *p*=1.6×10 ^-5^).

**Figure 5.**
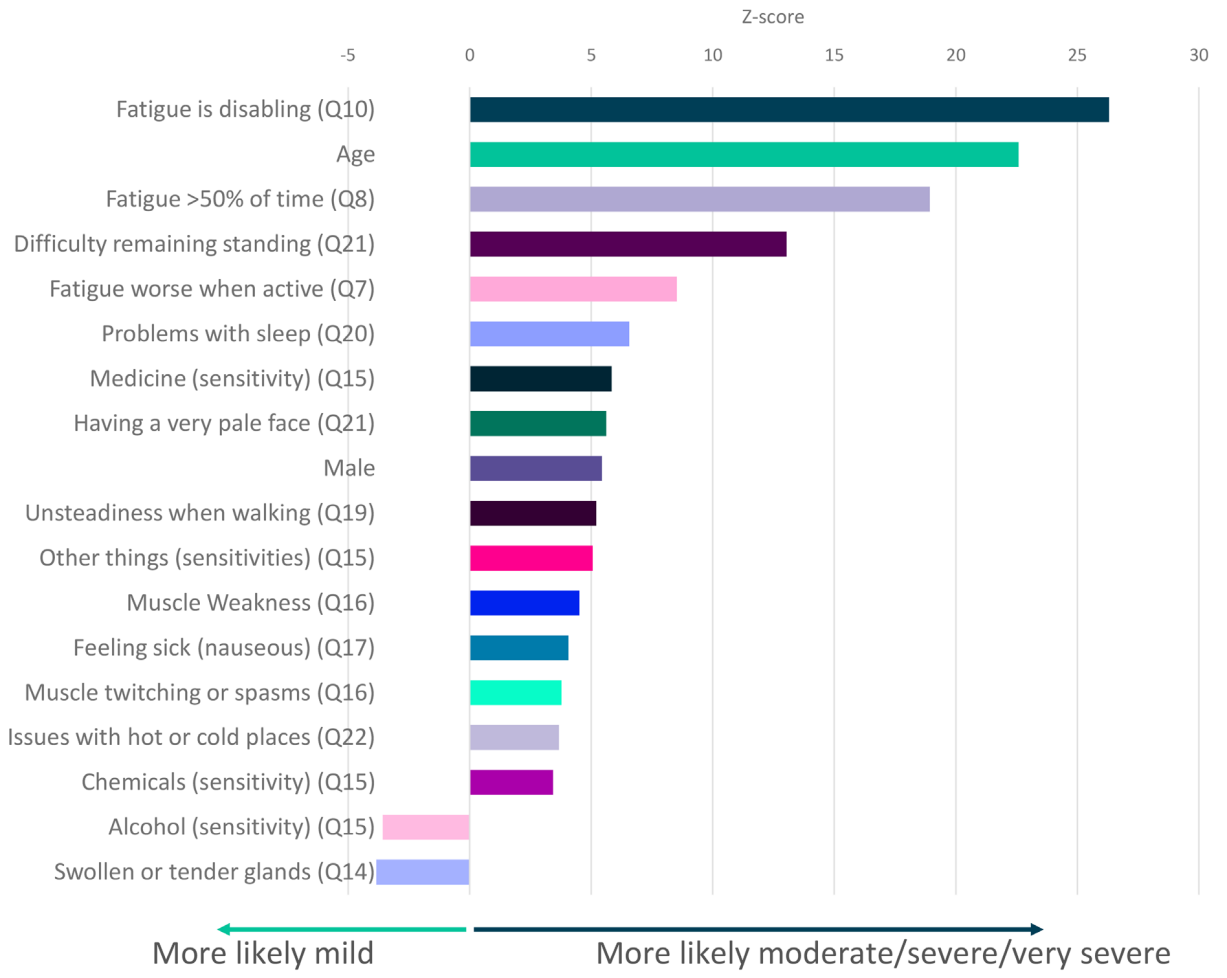
Questionnaire responses that significantly associate with ME/CFS symptom severity. Z-scores are shown for symptoms that significantly associate with severity ( *p*<0.05 after Bonferroni correction for 82 tests, including age and sex). Here severity is defined by self-report of moderate or severe or very severe symptoms versus self-report of mild symptoms (see Figure 3). Responses to questions 14 and 15 (Q14, Q15) are significantly associated with mild symptoms. Responses relate to DecodeME Questionnaire questions (e.g. question 10, Q10).

Testing all symptoms simultaneously with sex and age, showed strong and independent association between ME/CFS severity and 18 factors including fatigue, age, difficulty remaining standing, and sleep problems ( Figure 5). Finally, participants describing their illness as relapsing and remitting were significantly less likely to report their illness as moderate, severe or very severe than those reporting fluctuating symptoms ( *p*<2.2×10 ^-16^).

The type of infectious or non-infectious disease onset does not explain these strong and pervasive sex-biases because across the five onset types proportions of females were not significantly different (83.1%-84.5%; χ ^2^ = 1.707, df = 4, *p* = 0.79).

### ME/CFS characteristics by onset type

A feature that strongly distinguished among the five onset types was longevity of participants’ ME/CFS symptoms. Participants reporting an infection at onset were more likely to have had ME/CFS symptoms for over 10y than those reporting no infection at onset (67.0% vs. 43.6%). This is despite their similar ages (medians 50y and 46y, respectively).

The statistical significance of this difference is strong. When testing for association between those with an infection prior to ME/CFS onset and duration (<10y vs. >10 years since time of onset), age and sex, only association with duration was significant ( *p* = 4×10 ^-67^). This relative paucity of participants not reporting an infection prior to onset of their ME/CFS over 10y ago is unexpected, and not easily explained by historic variation in ME/CFS triggers because association with age was not significant in this analysis ( *p* > 0.05). When analysed separately, each onset type was not associated with participants’ sex, when including age and ME/CFS duration over 10y in the analysis.

**Figure 6.**
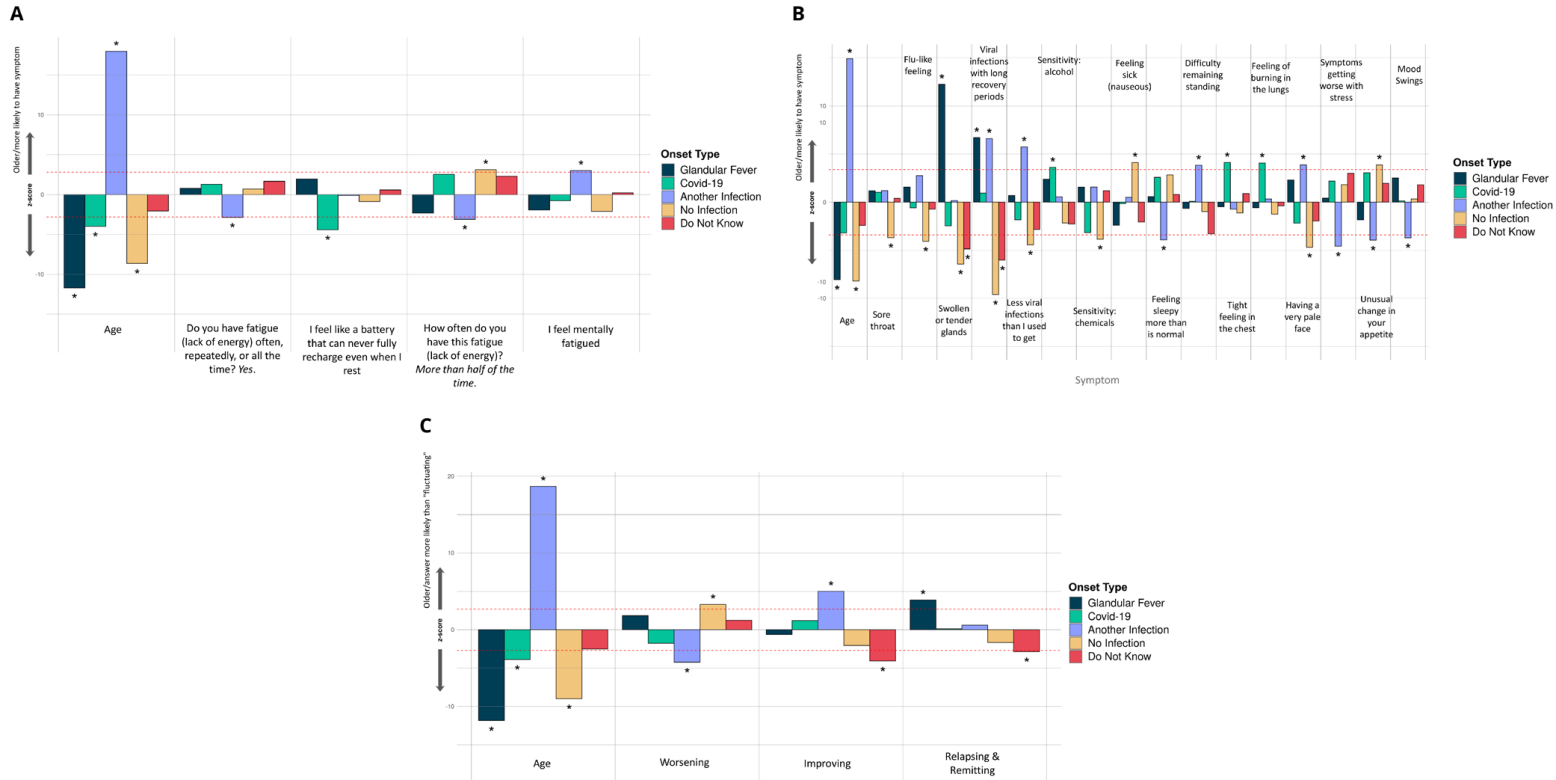
Associations of symptoms or age to 5 ME/CFS onset types: ( **A**) Fatigue symptoms (10 tests), ( **B**) Non-fatigue symptoms (74 tests), and ( **C**) Illness course descriptions (7 tests). These were considered in a logistic regression model of the form OnsetType ~ age + sex + symptoms/descriptions and an intercept. A covariate is only shown if it survived Bonferroni multiple testing correction ( *p*<0.05) per regression for one or more symptom/description. Significant associations are indicated with an asterisk (*); their Z-scores lie outside of non-significant values, bounded by the red dashed lines, after Bonferroni multiple testing correction. The z-score (Y-axis) is the effect-size estimate in standard deviation units.

There were significant differences between the 5 ME/CFS onset types and 4 fatigue symptoms ( Figure 6A), 16 other symptoms ( Figure 6B) and 3 different types of illness course ( Figure 6C). Those with glandular fever onset were significantly more likely than others to report swollen or tender glands and viral infections with long recovery periods within the last 6 months, and to experience relapsing and remitting symptoms (relative to ‘Fluctuating’, the majority response). Others with COVID-19 infection at ME/CFS onset preferentially reported a tight feeling in the chest, sensitivity to alcohol and a feeling of burning in the lungs. Participants with other types of infection onset more frequently reported feeling mentally fatigued, feeling fatigued less than half the time, and difficulties remaining standing, and less frequently reported feeling more sleepy than is normal, having worsening symptoms (relative to ‘Fluctuating’), unusual changes in appetite and mood swings.

Participants reporting an infectious onset (when compared to those who did not) were also significantly more likely to report: improving symptoms, relapsing/remitting, or recovered (relative to ‘Fluctuating’) symptoms, and less likely to report worsening symptoms (again, relative to ‘Fluctuating’). They were more likely, among other things, to report viral infections with long recovery periods, fewer viral infections than they used to get, and having a pale face. Other symptoms that were significantly more likely to be reported by participants without an identified infection at onset were fatigue more than half the time, reduced libido, and unusual changes in appetite. They were also less likely to report symptoms common during infection: flu-like feelings, and swollen or tender glands.

Those with an infection prior to onset of ME/CFS more frequently reported symptoms typical of infection in the last 6 months, whereas those reporting no infection at onset less frequently indicated these symptoms. This was unexpected because of the long time-lag between onset (mostly >10y ago) and participants’ recent questionnaire responses. Those with an infection prior to onset of ME/CFS frequently reported symptoms typical of infection in the last 6 months, whereas those reporting no infection at onset infrequently indicated these symptoms. Even though most participants report a long interval between their onset of ME/CFS (mostly >10y ago) and their recent symptoms characteristic of infection, our results cannot distinguish between whether these recent symptoms are a natural consequence of their ME/CFS onset, for example because of viral persistence in some individuals ^28^, or else they are independent of onset.

**Figure 7.**
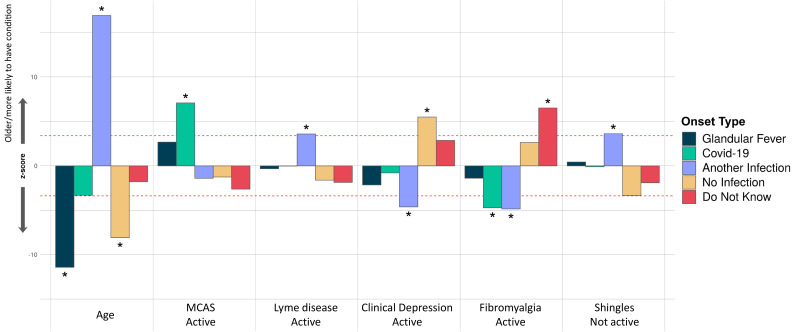
Associations of comorbidities or age to 5 ME/CFS onset types. Thirty-four comorbidities were considered in a logistic regression model of the form OnsetType ~ age + sex + comorbidities and an intercept. A covariate is only shown if it survived Bonferroni multiple testing correction ( *p*<0.05) for one or more onset type. Active and inactive comorbidities were considered independently: Active, if the condition has given symptoms in the past 6 months, or Inactive, if the condition has not given symptoms in the past 6 months, either because it has died down or treatment has controlled it. The z-score (Y-axis) is the effect-size estimate in standard deviation units.

In our last analysis, we tested for association between participants’ onset type and their comorbidities, age and sex. Only younger age, rather than any comorbidity, was significantly associated with glandular fever onset ( Figure 7). Among all onset types, only coronavirus disease 2019 (COVID-19 caused by SARS-CoV-2) infection was significantly associated active Mast Cell Activation Syndrome (MCAS), i.e. MCAS symptoms within the previous 6 months. COVID-19 related onset was also negatively associated with active fibromyalgia. Onset with another infection was positively associated with inactive Shingles or active Lyme disease, and negatively associated with fibromyalgia or clinical depression. Onset without reported infection at onset was significantly associated with recent clinical depression symptoms; and, onset with unknown infection status was significantly associated with active fibromyalgia as a comorbidity ( Figure 7).

## Discussion

DecodeME questionnaire responses show how people with ME/CFS do not form a single homogeneous group. Rather, large and significant differences exist among five ME/CFS onset types relating to symptoms, comorbidities and illness severity, as well as substantial differences between females and males. Studies involving hundreds of participants previously concluded that ME/CFS exhibits few sex differences in illness patterns ^29, 30^. Smaller studies indicated older age as associated with greater ME/CFS symptom severity, but other studies found no such association (reviewed in 12, 31). These previously limited cohort sizes did not permit comprehensive analysis. In a previous study, three symptoms were reported significantly more often by females than males: fever, swollen glands, and sore throat ^30^. In our study, we replicated these findings, and found a further 59 of 80 ME/CFS symptoms that are also female-biased. Our analyses additionally found 61 symptoms biased towards younger age, with only 5 biased towards older age.

The raw number of symptoms may not be meaningful, however, as symptoms can be overlapping, and people with ME/CFS may, over time, pace sufficiently to avoid triggering some symptoms or may begin to describe their symptoms with fewer labels, particularly when interventions are not available to treat each symptom effectively. Indeed, rather than younger participants reporting increased severity, we found that being female, older and over 10y from onset are all risk factors for ME/CFS severity.

Despite its large cohort size (N=17,074), extensive community reach and use of paper, as well as electronic, questionnaires, the analysis presented here – of the December 2022 DecodeME data freeze – has three main limitations. First, recruitment is restricted to participants over the age of 16y, which limited investigation of paediatric ME/CFS. Second, when asking participants if they were diagnosed by a health professional we did not require clinical confirmation of reported answers. Nevertheless, our extensive engagement with participants and the internal consistency of their responses encourage us to believe that questionnaire answers have been given in good faith, noting that inconsistent responses may result from respondents’ ME/CFS symptoms including the cognitive dysfunction of ‘brain fog’. Thirdly, regrettably DecodeME has not yet been successful in recruiting proportionately from minoritised groups. There is little consensus on whether ME/CFS prevalence differs among these and other groups ^32^. Other recruitment and representativeness biases are also possible, as with all research cohorts.

A previous study indicated that ME/CFS onset type associates with severity ^33^ although this was not replicated by our larger study. Instead, we identified large numbers of comorbidities and symptoms that are each more likely to be reported by participants with a specific onset type. We report significant associations to five onset types derived from participants’ responses to the question ‘Did you have an infection when, or just before, your first ME/CFS symptoms started?’:

1. ‘ **Yes, glandular fever**’ (17%): These participants were more likely to report swollen or tender glands and viral infections with long recovery periods, and to experience relapsing and remitting symptoms.
2. ‘ **Yes, COVID-19**’ (2%): These participants were more likely to report having Mast Cell Activation Syndrome, a tight feeling in the chest or a burning feeling in the lungs. Mast cell activation symptoms are prevalent in Long-COVID ^34^ but this condition is rarely diagnosed in people with ME/CFS ^35^ although perhaps because only recently have MCAS diagnostic criteria been defined ^36^.
3. ‘ **Yes, another infection**’ (44%): These participants were more likely to be mentally fatigued, to report viral infections needing long recovery periods, and to have had Shingles in the past or symptomatic Lyme disease in the last 6 months. They were also less likely than others to report active clinical depression or fibromyalgia. Over 100 types of infections have been reported to occur at ME/CFS onset ^11^.
4. ‘ **No**’ (i.e. no infection at onset; 16%): These participants were more likely to report fatigue more than half of the time, to feel nauseous, and to have recent clinical depression symptoms.
5. ‘ **Don’t know**’ (21%): These were more likely to report fibromyalgia as a comorbidity, and less likely to report cold or flu-like, improving or relapsing and remitting symptoms.

These onset types reveal differences amongst those with ME/CFS regarding their symptoms and comorbidities ( Figure 4). However, these distinctions are not absolute. For example, those reporting no infection at onset (Type 4, above) are not cleanly distinguished from all others by active clinical depression. Rather, they were the only onset type that was more likely to report this diagnosis (25.4%) than all other participants were (19.6%). Similarly, Type 3 contains a higher proportion (9.4%) of those who report inactive shingles, than all other participants (7.3%). Shingles is caused by reactivation of latent varicella-zoster virus (a herpesvirus). People with herpes zoster infection are known to have a significantly higher risk of ME/CFS up to at least 6 years ^37^ fuelling speculation that varicella-zoster virus infection is a cause of ME/CFS that may be prevented by vaccination. 2.5% of ME/CFS cases have been attributed to varicella-zoster virus infection ^11^. We note that among those reporting no infection prior to onset (Type 4) some may have developed ME/CFS secondary to an infection without an obvious acute phase, such as can occur with Epstein-Barr virus ^38^. However, we are unable to test this hypothesis here.

ME/CFS’ poor long-term prognosis, its severe symptoms – especially for older females, its profound impact on the quality of life of people with ME/CFS and family members ^9, 27^, and its high population prevalence (>0.2%) ^1^ present formidable healthcare and research challenges. Considering that 63% of DecodeME participants reported an infection prior to onset, any vaccination against the major infectious agents triggering ME/CFS, including Epstein-Barr virus ^39^, SARS-CoV-2 ^40^ and influenza viruses ^41^ may help reduce ME/CFS incidence in the future, especially for individuals more susceptible to severe disease, or those more likely to be exposed to the infectious agents.

Formal investigation of each onset type’s clinical significance is now warranted. To give hope to each of the millions of people worldwide affected by ME/CFS that effective therapeutic interventions will be found within their lifetime, the research community and policy-makers will need to give sustained focus on disease classification and aetiology. It is for this reason that DecodeME is seeking to identify genetic factors causal of altered ME/CFS risk ^14^ and will do so for infectious versus non-infectious onset participants separately and combined, if final recruitment numbers allow. Recruitment to the DecodeME study is ongoing.

## Data Availability

Anonymised data allowing investigation of this study’s consented data are available to researchers by managed access via a Data Access Committee, https://www.decodeme.org.uk/faqs/who-will-be-able-to-use-my-data-and-sample/. This committee consists of a scientist, a patient and a charity representative who strictly control access to the data. DecodeME’s anonymised and consented data are only shared with studies that meet high standards and whose academic or industrial researchers agree to treat its data with respect and to keep it secure.
