# Typing myalgic encephalomyelitis by infection at onset: A DecodeME study

**DOI:** 10.3310/nihropenres.13421.2

**Published:** 2023-07-04

**Authors:** Andrew D. Bretherick, Simon J. McGrath, Andy Devereux-Cooke, Sian Leary, Emma Northwood, Anna Redshaw, Pippa Stacey, Claire Tripp, Jim Wilson, Sonya Chowdhury, Isabel Lewis, Øyvind Almelid, Sumy V. Baby, Tom Baker, Hannes Becher, Thibaud Boutin, Malgorzata Clyde, Diana Garcia, John Ireland, Shona M. Kerr, Ewan McDowall, David Perry, Gemma L. Samms, Veronique Vitart, Jareth C. Wolfe, Chris P. Ponting

**Affiliations:** 1 MRC Human Genetics Unit, The University of Edinburgh, Edinburgh, Scotland, EH4 2XU, UK; 2 Pain Service, Ninewells Hospital, NHS Tayside, Dundee, Scotland, DD1 9SY, UK; 3 c/o DecodeME, MRC Human Genetics Unit, The University of Edinburgh, Edinburgh, Scotland, EH4 2XU, UK; 4 42 Temple Street, Keynsham, Action For ME, Bristol, England, BS31 1EH, UK

**Keywords:** Myalgic encephalomyelitis, Post-viral syndrome, Post-exertional malaise, Sex-bias, Sub-types

## Abstract

**Background:**

People with myalgic encephalomyelitis/chronic fatigue syndrome (ME/CFS) experience core symptoms of post-exertional malaise, unrefreshing sleep, and cognitive impairment. Despite numbering 0.2-0.4% of the population, no laboratory test is available for their diagnosis, no effective therapy exists for their treatment, and no scientific breakthrough regarding pathogenesis has been made. It remains unknown, despite decades of small-scale studies, whether individuals experience different types of ME/CFS separated by onset-type, sex or age.

**Methods:**

DecodeME is a large population-based study of ME/CFS that recruited 17,074 participants in the first 3 months following full launch. Detailed questionnaire responses from UK-based participants who all reported being diagnosed with ME/CFS by a health professional provided an unparalleled opportunity to investigate, using logistic regression, whether ME/CFS severity or onset type is significantly associated with sex, age, illness duration, comorbid conditions or symptoms.

**Results:**

The well-established sex-bias among ME/CFS patients is evident in the initial DecodeME cohort: 83.5% of participants were females. What was not known previously was that females tend to have more comorbidities than males. Moreover, being female, being older and being over 10 years from ME/CFS onset are significantly associated with greater severity. Five different ME/CFS onset types were examined in the self-reported data: those with ME/CFS onset (i) after glandular fever (infectious mononucleosis); (ii) after COVID-19 infection; (iii) after other infections; (iv) without an infection at onset; and, (v) where the occurrence of an infection at or preceding onset is not known. Among other findings, ME/CFS onset with unknown infection status was significantly associated with active fibromyalgia

**Conclusions:**

DecodeME participants differ in symptoms, comorbid conditions and/or illness severity when stratified by their sex-at-birth and/or infection around the time of ME/CFS onset.

## Plain English summary

Myalgic Encephalomyelitis / Chronic Fatigue Syndrome (ME/CFS) is a chronic disease that affects an estimated 250,000 people in the UK. Its defining symptom is post-exertional malaise, an excessive delayed worsening of symptoms following even minor physical or mental exertion. For those with it, ME/CFS means disability and poor quality of life.

DecodeME is a research study which is looking for DNA differences between people with ME/CFS and people without any health problems. People with ME/CFS who take part in DecodeME complete a questionnaire that assesses their symptoms and whether they will then be invited to donate a DNA sample. This paper analyses the answers to this questionnaire; we will publish results of the DNA analysis separately.

So far, more than 17 thousand people with ME/CFS have completed the DecodeME questionnaire. Their answers help us to address the question: “Are there different types of ME/CFS linked to different causes and how severe it becomes?”

Results show that people with ME/CFS do not form a single group reporting similar symptoms and additional medical conditions. Instead, participants who had an infection at the start of their ME/CFS reported a different pattern of symptoms and conditions compared to those without an infection.

It is well known that most people with ME/CFS are females. What was not clear previously was that females tend to have more additional health conditions. Also, being female, being older and being over 10 years from ME/CFS onset all make it more likely that someone is more severely affected by their ME/CFS.

These findings could indicate that by studying people with different ME/CFS onset-types separately – rather than analysing all people with ME/CFS together – it will be easier to understand what is going wrong.

## Introduction

Myalgic encephalomyelitis / chronic fatigue syndrome (ME/CFS) is a chronic multisystem disorder that affects an estimated 0.2–0.4% of the UK population ^1, 2^. Its core symptoms are post-exertional malaise, pain, fatigue, unrefreshing sleep, cognitive impairment and/or orthostatic intolerance that may each change across the life-course ^3^. Many people with ME/CFS report an infectious episode prior to their initial symptoms. Up to 10% of people with glandular fever (also known as infectious mononucleosis) are eventually diagnosed with ME/CFS ^4, 5^, with similar fractions of people with Ross River virus or *Coxiella burnetii* infections also developing ME/CFS ^4^. Long COVID, whose symptoms can overlap those of ME/CFS, appears to arise at a similar rate after infection with severe acute respiratory syndrome coronavirus-2 (SARS-CoV-2) ^6, 7^. Onset of ME/CFS can also occur without report of infection ^8^. Pathogenesis is unknown, and effective treatment is not available. In one study, the health-related quality of life for people with ME/CFS was worse than 20 other conditions compared, including breast, prostate, colon or lung cancer, type I or II diabetes, stroke, multiple sclerosis and schizophrenia ^9^.

One priority from a 2022 priority setting exercise facilitated by the James Lind Alliance ^10^ was “Are there different types of ME/CFS linked to different causes and how severe it becomes? Do different types of ME/CFS need different treatments or have different chances of recovery?” To address this question, we took advantage of questionnaire data from DecodeME, a study launched in the UK in September 2022. Before the end of the year, over 17,000 people with a ME/CFS diagnosis from a health professional, and at least 16 years (y) old, had been recruited and completed the study questionnaire.

Over many decades, ME/CFS studies have addressed similar questions using symptom data for tens or hundreds of participants recruited using various inclusion and exclusion criteria ^8, 11, 12^. However, they remain inconclusive on whether different ME/CFS types exist and whether symptoms are sex-biased. The DecodeME project provided a unique opportunity to perform adequately-powered analyses for detecting differences within a single large ME/CFS cohort, under an assumption that ME/CFS type is delineated by onset type.

## Methods

### Patient and Public Involvement

The DecodeME project grew out of the UK ME Research Collaborative (MERC), formerly known as the CFS/M.E. Research Collaborative or CMRC, which was first established in 2013. The MERC includes people with ME/CFS and carers within a Patient Advisory Group (PAG). As the project evolved in 2018–19, Patient and Public Involvement (PPI) was embedded in every discussion and workshop, resulting in the project becoming a co-production with its grant proposal, aims and outcomes being decided by researchers and PPI equally. In 2020, PPI Steering Group members were selected from across diverse charities and organisations, and for their breadth of experience. The project’s name was suggested and decided by PPI Steering Group members. In DecodeME, PPI representatives serve on each of its delivery groups, lead on marketing and communication (including social media), and contribute the majority (two of three) members of the decision-making body, the Management Group. People with lived experience of ME/CFS led the co-creation of a new DecodeME questionnaire. This resulted in substantial improvements in comprehension and accuracy compared to initial drafts and reduced the burden on participants, thereby boosting recruitment.

DecodeME’s genetics question (“What, if any, significant genetic differences are there between people with — and those without — ME/CFS?") was identified as a priority first by the MERC and its PAG, before being confirmed as a priority by a wider section of the patient community in the results of the Priority Setting Partnership for ME/CFS ^10^. Established participant selection criteria were further refined with PPI throughout. PPI members, through their profound understanding of ME/CFS phenotypes, triggers, severity, symptom range, comorbidities and more, have improved the study’s adherence to our chosen case definition and thus further assured the relevance of genetic associations to ME/CFS lived experience.

A substantial minority (16 of 41; 39%) of volunteer participants who trialled an initial paper questionnaire experienced difficulties when answering its questions, missing out questions, marking too many answers or adding their own responses. We then created a substantially revised version with which fewer participants found difficulties (88 of 470; 19%). This revised version again implemented Canadian Consensus criteria (CCC) and IOM/NAM criteria ^3, 13^ as well as criteria introduced in response to peer reviewers’ comments on the grant application. A total of 14,789 (86.6%) participants met CCC and/or IOM/NAM criteria. The final DecodeME questionnaire captures participants’ age and sex at birth, their ME/CFS illness severity, duration, course, associated symptoms and co-occurring conditions, and whether they experienced an infection around the time of their first ME/CFS symptoms occurring. The questionnaire contains 10 questions on personal information and 29 questions on symptoms; it additionally allows participants to indicate whether a health professional has diagnosed them with any of 34 conditions. Symptom questions allowed multiple choice, others require single answers. This questionnaire is freely available from the DecodeME website. As a co-production, PPI members advised and helped to create both our recruitment strategy and recruitment materials. Further description of DecodeME’s recruitment methods and PPI aspects can be found elsewhere ^14^. Before study launch, public awareness of DecodeME was enhanced using regular podcasts, webinars, blog posts and media interviews. These media channels will be used by PPI members and scientists to disseminate results to the international ME/CFS community. PPI team members maintain extensive input into reporting of the results of the questionnaire (including in this article), providing greater understanding and context, and ensuring accessibility. Our genome-wide association study and analysis plans were co-created by researchers and PPI members.

The DecodeME study was reviewed and given a favourable opinion by the North West – Liverpool Central Research Ethics Committee (21/NW/0169). Potential participation bias due to internet use was mitigated by providing a paper questionnaire and providing participants with assistance in completing their online questionnaires. Team members were available to answer phone calls and emails during working hours.

### Cohort

Diverse methods used to identify potential participants are detailed in the open access DecodeME Study Protocol publication ^14^. Between its full launch date of September 12, 2022 and a data freeze performed on December 19, 2022, DecodeME recruited 17,074 female or male participants who self-reported a diagnosis of ME, CFS, ME/CFS or CFS/ME by a health professional and consented to participate. It is this cohort that we analyse here. All participants were aged 16y or older and completed a questionnaire either online (98.1%) or with a paper version (1.9%). Participants were asked for their sex assigned at birth, how long they had experienced ME/CFS symptoms, and information about 34 conditions: “If a health professional has ever told you that you had any of the conditions below, please select all that apply. If the conditions don’t apply to you, please do not select any box.” Participants indicated whether each condition was Active (“If the condition has given you symptoms in the past 6 months”) or not active (“If the condition has not given you symptoms in the past 6 months, either because it has died down or treatment has controlled it”). They were also asked about 9 fatigue- and 73 non-fatigue symptoms: “In the last 6 months, have you had any of the symptoms below often, repeatedly, or constantly? Please mark any that apply. If none apply, leave all the boxes blank.” Respondents were asked: “How severe is your illness?” with answer options matching severity definitions from the UK’s National Institute for Health and Care Excellence (NICE) guidelines (2021). Severity categories were consistent with participants’ reports of their comorbidities and symptoms ( **Results**). Participants indicated the duration of their ME/CFS illness by selecting from a set of predefined ranges, for example between 5 and 10 years, or over 10 years, since onset of symptoms. Questionnaire responses from participants who both consented to participate and self-reported being given a diagnosis of ME, CFS, ME/CFS or CFS/ME by a health professional (as of 19 December 2022) were analysed. Only those whose sex assigned at birth was male or female were analysed due to insufficient numbers of other identities. Participant ages were as of 19 December 2022. Further analyses of questionnaire and genotype data will be undertaken for the full DecodeME cohort once the recruitment phase of the project is completed.

***Significance testing.*** Logistic regression analyses were used to evaluate the relationship between various predictor variables (e.g. age or sex or comorbidities) and a binary outcome (e.g. symptom or onset type). For this we used the glm function in R version 4.2.2. Only *p*-values surviving Bonferroni correction for multiple tests (nominal *p*-value, here 0.05, divided by the number of tests per analysis) are shown. To address the question “What self-reported symptoms are associated with sex and/or age?” for each of 80 symptoms we used the linear model: Symptom ~ age + sex + intercept – Figure 4. To address “What symptoms are associated with severity?” for 80 symptoms we used the model: Severity ~ age + sex + symptoms + intercept – Figure 5. To address the questions “What onset types are associated with each of 8 fatigue symptoms ( Figure 6A) or 72 non-fatigue symptoms ( Figure 6B) or 5 illness courses ( Figure 6C)?” we used the model: OnsetType ~ age + sex + symptoms + intercept. To address “What onset types are associated with 34 comorbidities (active and inactive)?” we used the model: OnsetType ~ age + sex + comorbidities + intercept – Figure 7. For the relevant analyses, severity was coded as mild versus others (i.e. moderate or severe or very severe) – Figure 5; 5 illness courses were compared with ‘Fluctuating’, the majority response – Figure 6C.

## Results

**Figure 1.**
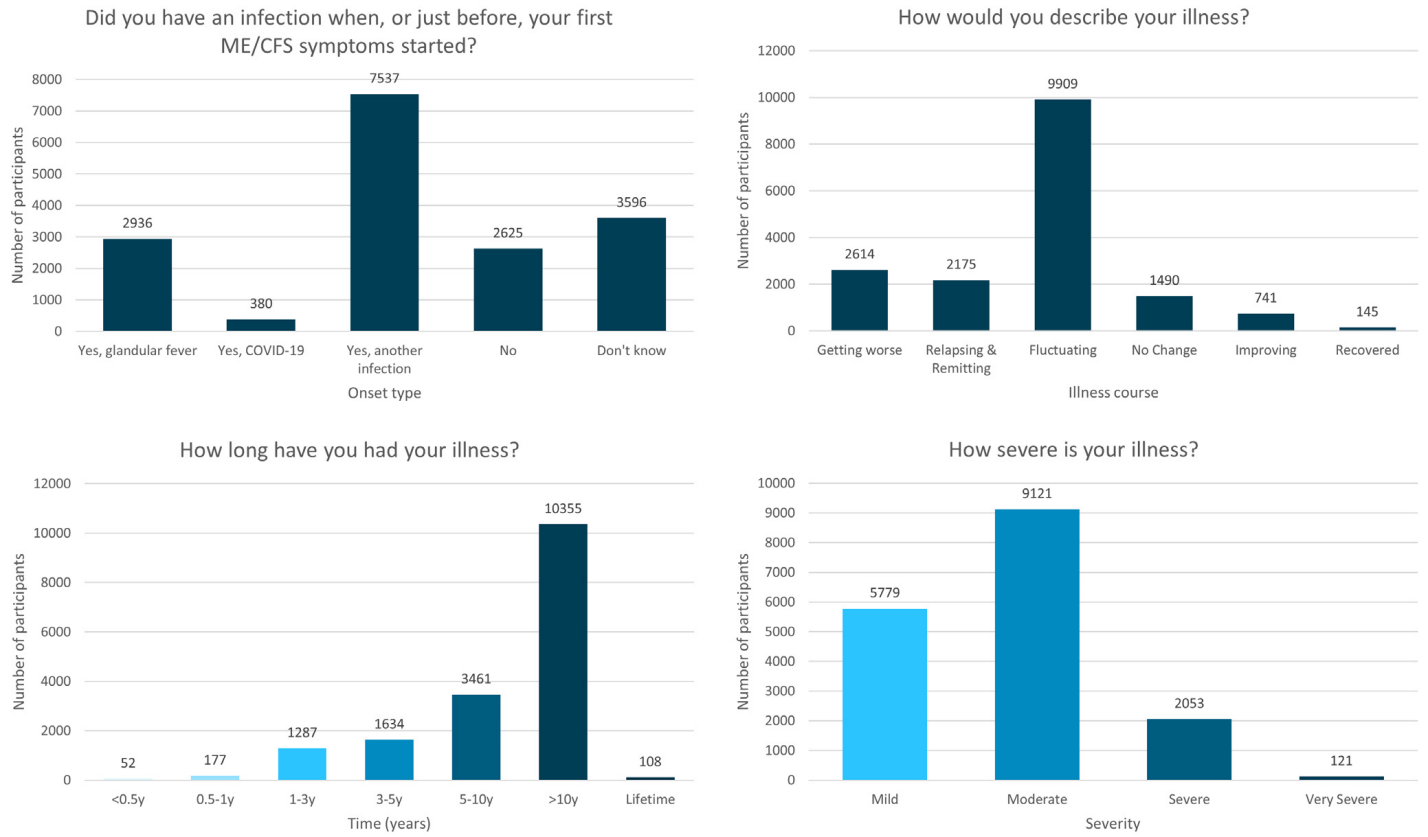
Onset type, illness course, duration of illness and severity of the DecodeME cohort. Numbers of DecodeME participants reporting whether they had an infection prior to ME/CFS onset (top left) as well as their illness’ course (top right), duration (bottom left) and severity (bottom right; *n* = 17,074 participants).

This initial DecodeME cohort contained 17,074 participants (83.5% females) whose median age was 49y (interquartile range [IQR] 37y-59y). Male participants tended to be older than females (median 52y [IQR=40y-63y] and 48y [IQR=37y-59y] respectively; p< 2.2×10 ^-16^, Wilcoxon rank sum test). Only 3.3% ( *n*=557) of 17,074 participants did not self-report their ethnicity as White, far fewer than the 18.3% in England and Wales who identify as non-White ( https://www.ethnicity-facts-figures.service.gov.uk/). Most DecodeME participants’ severity levels are categorised as Mild or Moderate, but Severe and Very Severe individuals are also represented ( Figure 1).

Two-thirds (n=10,853; 63.6%) reported an infectious onset to their symptoms ( Figure 1), such as glandular fever (n=2,936; 17.2%), COVID-19 (n=380, 2.2%) or another infection ( *n*=7,537; 44.1%). However, only 68% ( *n*=2,009), 51% ( *n*=192) and 26% ( *n*=1,953) respectively of respondents with these potential triggers reported a positive laboratory test confirming the infectious agent.

Over half (58.0%; *n*=9,909) indicated that their ME/CFS is “Fluctuating (my symptoms vary day to day but don’t go away)”, 12.7% ( *n*=2,175) describe their symptoms as “Relapsing and remitting (good periods with no symptoms alternating with symptomatically bad periods)” and 15.3% ( *n*=2,614) indicate their symptoms are “Getting worse” ( Figure 1).

Most (61.3%; *n*=10,463) participants have had ME/CFS for over 10y, and 81.6% ( *n*=13,924) over 5y ( Figure 1). Together, study participants have experienced over 1.3x10 ^5^ years of ME/CFS symptoms.

**Figure 2.**
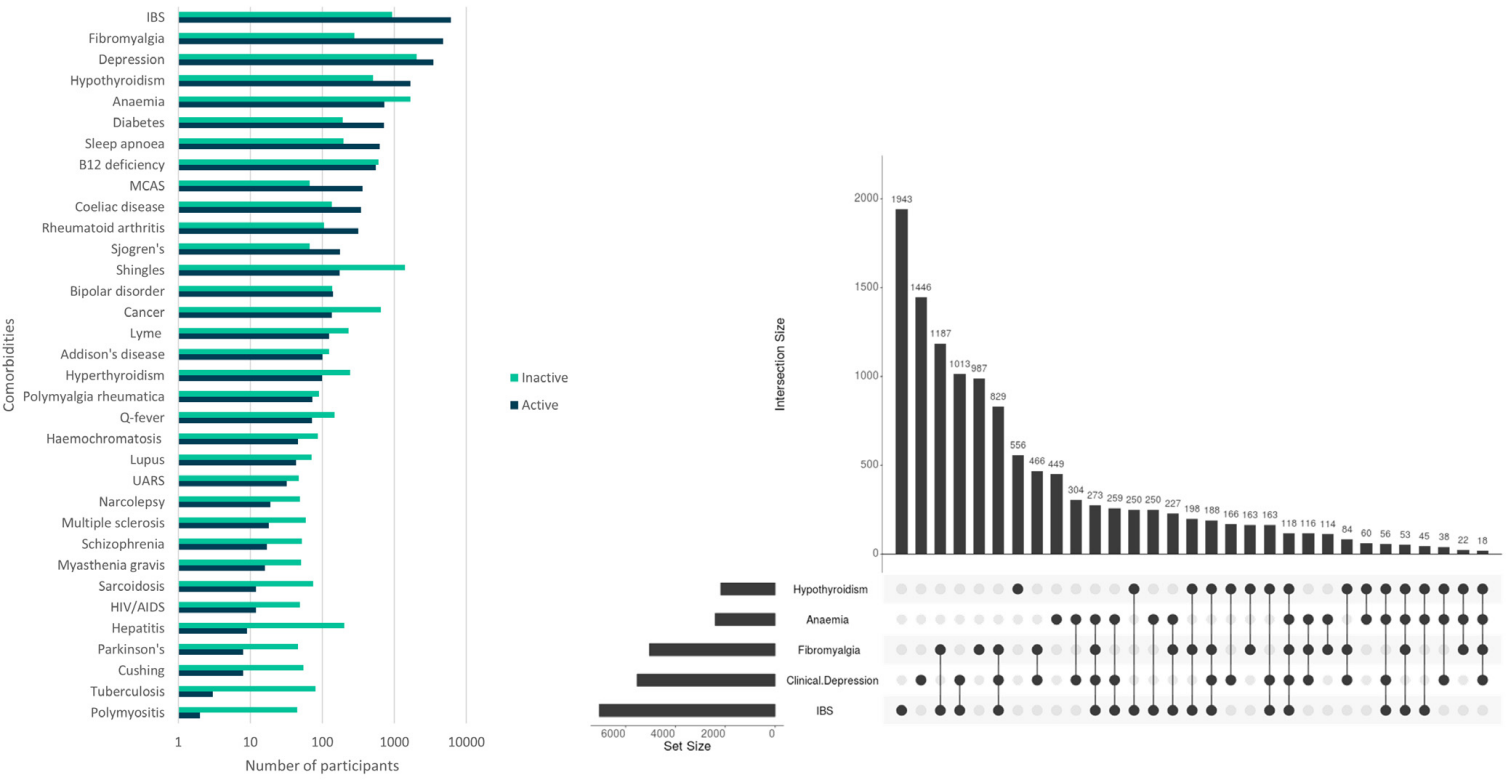
Numbers of DecodeME participants reporting conditions co-occurring with ME/CFS (comorbidities); total, 17,074 participants. In ( **A**) numbers are shown in log _10_-scale and those with active or inactive comorbidities are indicated in blue or green, respectively. The UpSet plot ^16^ ( **B**) shows numbers of participants with five conditions that most frequently co-occur with ME/CFS. These either co-occur together with others (indicated by filled circles linked by lines) or else separately (filled circles not linked by lines).

50.6% ( *n*= 8,637) of participants (all with self-reported ME/CFS diagnosed by a health professional) reported two or more comorbid conditions, most commonly irritable bowel syndrome (IBS; 41.3%; *n*=7,052), clinical depression (32.4%; *n*=5,537) and fibromyalgia (29.5%; *n*=5,043), anaemia (14.1%; *n*=2,402) and hypothyroidism (12.8%; *n*=2,178) ( Figure 2). Fibromyalgia and IBS occur together with ME/CFS for 18.0% ( *n*=3,073) of participants ( Figure 2B). 22.6% ( *n*=3,865) report no comorbidities.

**Figure 3.**
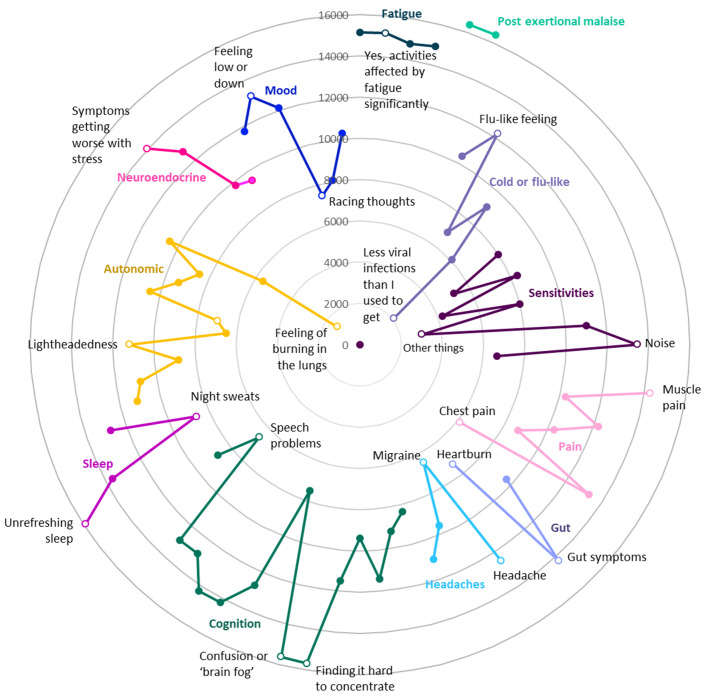
Numbers of DecodeME participants reporting symptoms (Radar chart); total, 17074 participants. Most frequently reported symptoms are furthest from these circles’ centre. Twelve different groups of questions are indicated in separate colours; for each symptom group, the most and least frequently reported symptoms are listed and indicated as unfilled circles. With reference to the DecodeME questionnaire ( www.decodeme.org.uk/app/uploads/2022/08/DecodeME-Questionnaire.pdf) the questions (Q) are, clockwise: Fatigue (Q8-answer 3 [Q8-3], Q9-3, Q3-1, Q10-1), Post-exertional malaise (PEM, Q12-1 AND Q13-1), Cold or flu-like (Q14-4, -2, -1, -5, -6, -3), Sensitivities (Q15-1, -2, -3, -5, -7, -9, -4, -6, -8), Pain (Q16-4, -6, -5, -3, -2, -7, -1), Gut (Q17-1, -2, -3), Headaches (Q18-1, -4, -2, -3), Cognition (Q19-15, -7, -8, -9, -12, -3, -1, -2, -6, -10, -5, -13, -4, -14, -11), Sleep (Q20-4, -3, -2, -1), Autonomic (Q21-3, -6, -11, -10, -9, -4, -2, -1, -5, -7, -12, -8), Neuroendocrine (Q22-3, -1, -2, -4), and Mood (Q23-2, -3, -1, -5, -6, -4).

DecodeME participants’ most frequent symptom is post-exertional malaise, a cardinal symptom of ME/CFS ^3^, followed by unrefreshing sleep, confusion or brain fog, fatigue, muscle pain and gut symptoms ( Figure 3). Almost all answered that once they had exceeded their energy limit their change in symptoms lasts “a long time, which can be more than 24 hours” (97.5%; *n*=16,649) and agreed that their fatigue affected them both physically and mentally (96.2%; *n*=16,433). For 88.7% ( *n*=15,142), their fatigue occurs more than half of the time and 87.3% ( *n*=14,921) report their fatigue as disabling.

**Figure 4.**
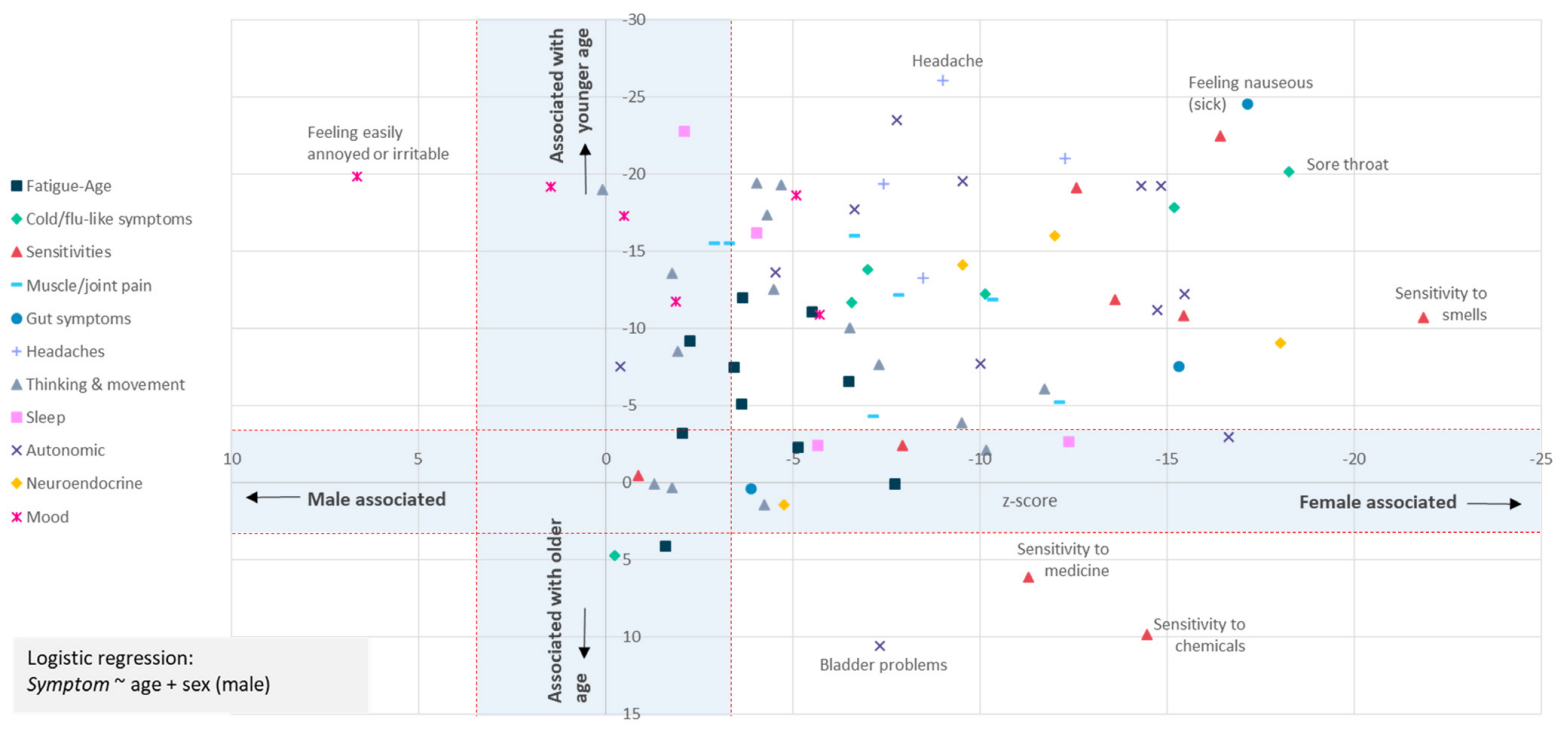
Most symptoms are strongly associated with female sex at birth and younger age. The question asked was: “In the last 6 months, have you had any of the symptoms below often, repeatedly, or constantly? Please mark any that apply.” Sex-biased (X-axis) and/or Age-biased (Y-axis) associations in a logistic regression analysis ( *Symptom* ~ age + sex + intercept) are shown as data points. Data points within the blue-shaded areas are not significant after accounting for 82 tests ( *p*<0.05/82, or |Z|<3.427. Only one symptom (“Feeling easily annoyed or irritable”) was male-biased; 3 symptoms (sensitivities to chemicals or medicine, or bladder problems) were associated with older age. Results for 80 symptoms are shown.

**Figure 5.**
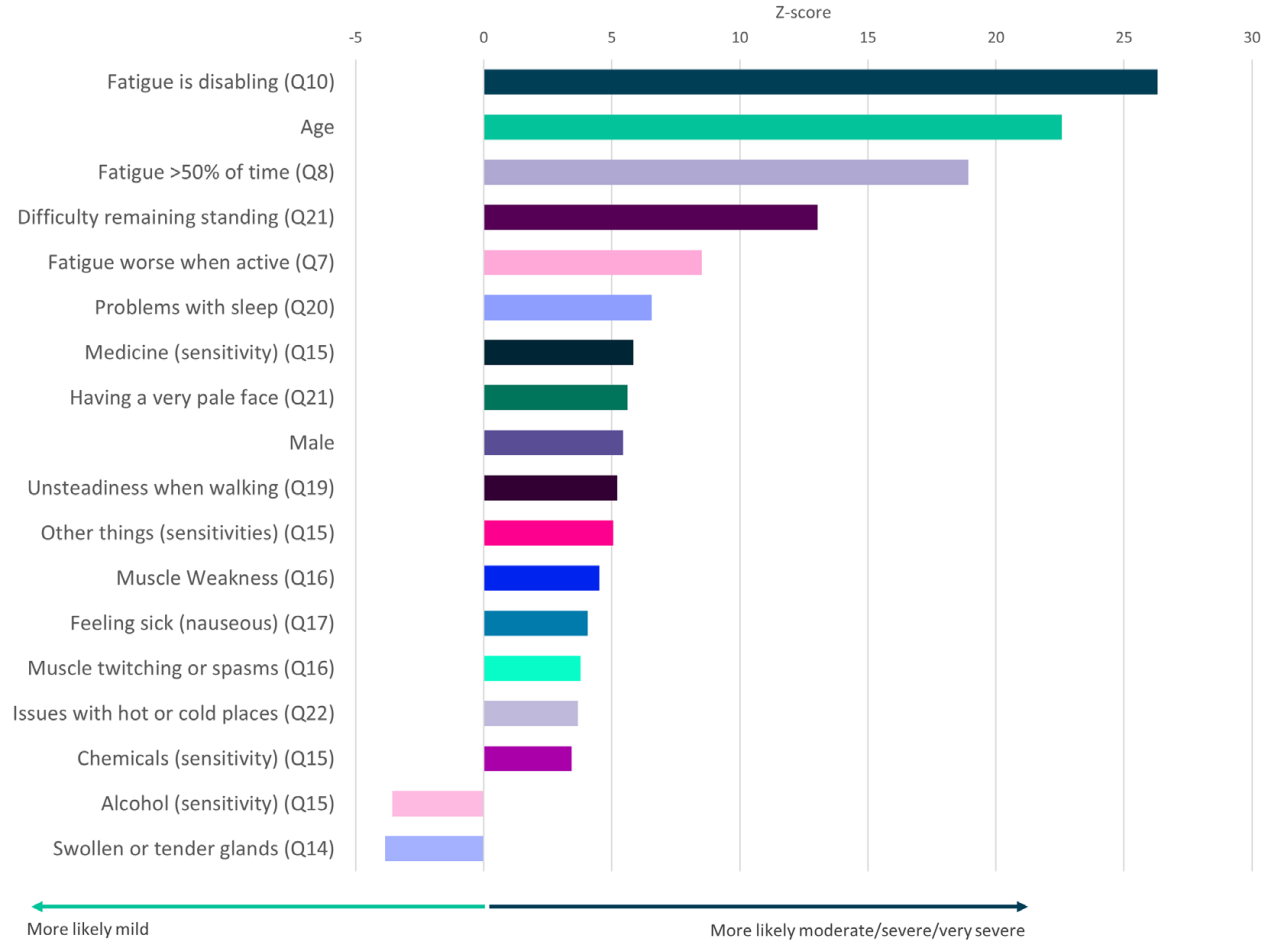
Questionnaire responses that significantly associate with ME/CFS symptom severity. Z-scores are shown for symptoms that significantly associate with severity ( *p*<0.05 after Bonferroni correction for 82 tests, including age and sex). Here severity is defined by self-report of moderate or severe or very severe symptoms versus self-report of mild symptoms (see Figure 1). Responses to questions 14 and 15 (Q14, Q15) are significantly associated with mild symptoms. Responses relate to DecodeME Questionnaire questions (e.g. question 10, Q10).

**Figure 6.**
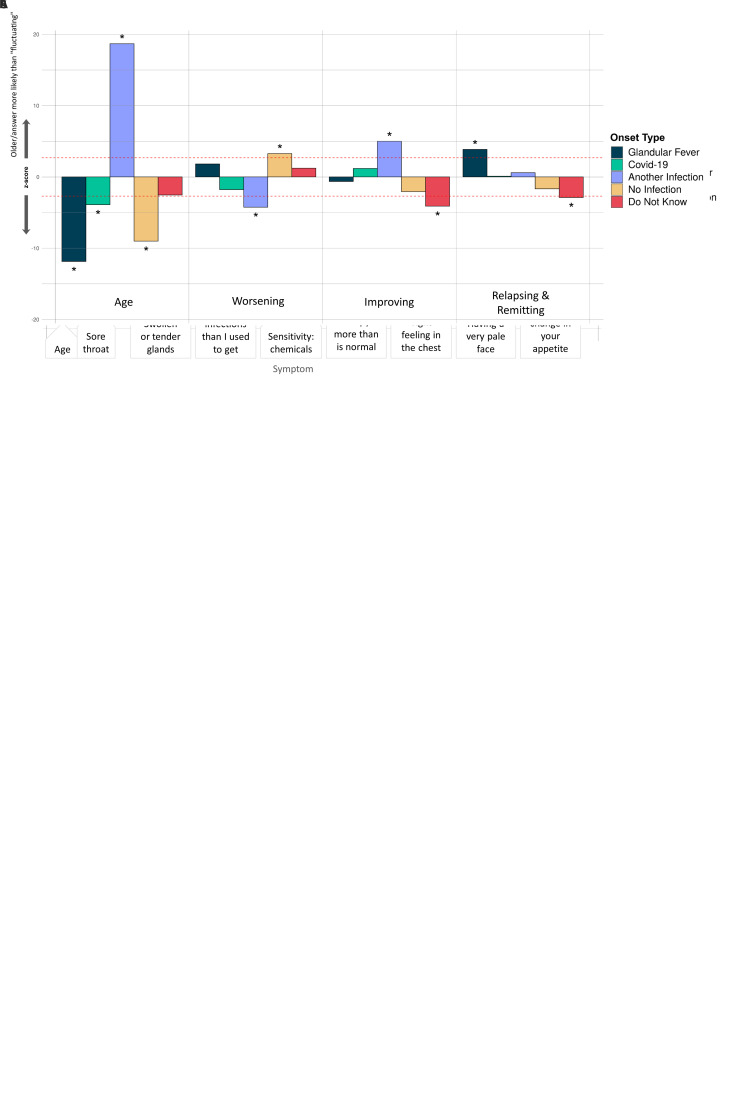
Associations of symptoms or age to 5 ME/CFS onset types: ( **A**) Fatigue symptoms (10 tests), ( **B**) Non-fatigue symptoms (74 tests), and ( **C**) Illness course descriptions (7 tests). These were considered in a logistic regression model of the form OnsetType ~ age + sex + symptoms/descriptions and an intercept. A covariate is only shown if it survived Bonferroni multiple testing correction ( *p*<0.05) per regression for one or more symptom/description. Significant associations are indicated with an asterisk (*); their Z-scores lie outside of non-significant values, bounded by the red dashed lines, after Bonferroni multiple testing correction. The z-score (Y-axis) is the effect-size estimate in standard deviation units.

**Figure 7.**
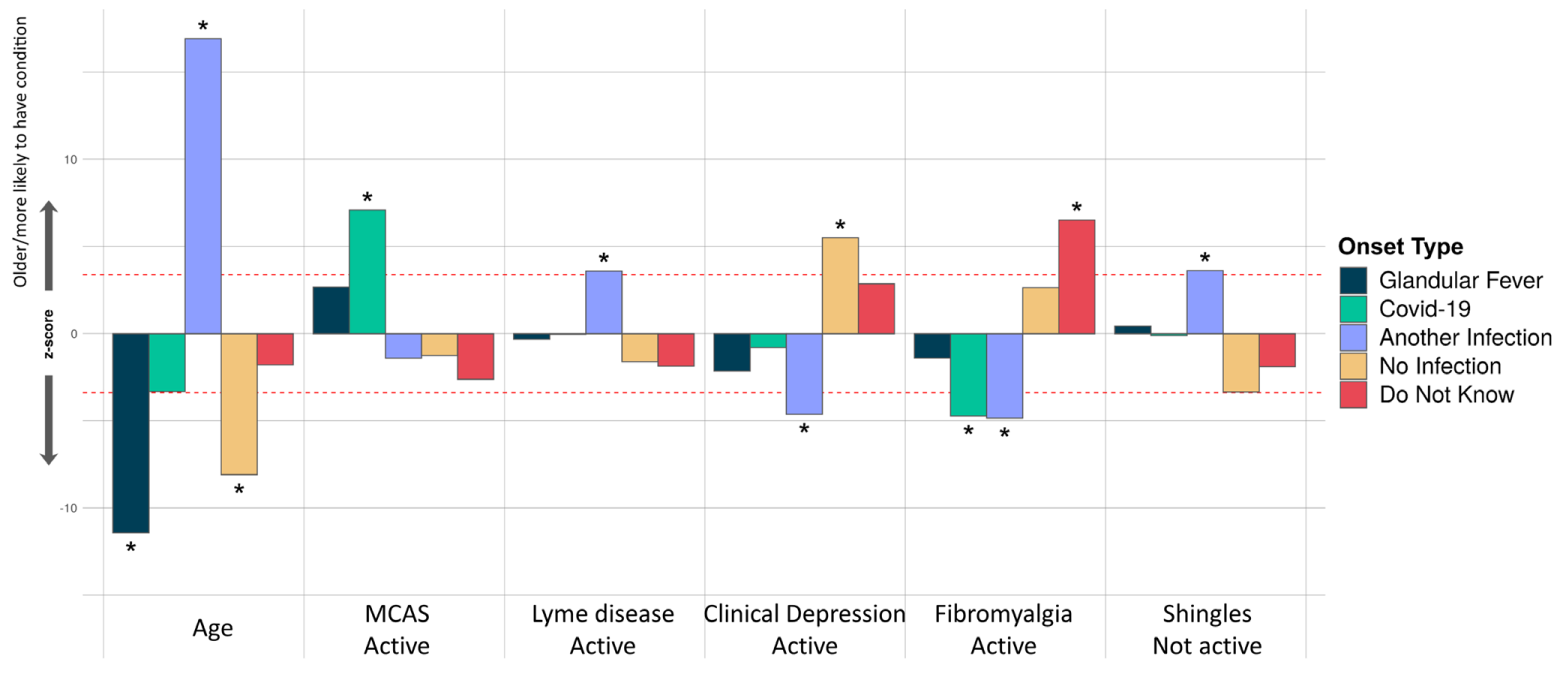
Associations of comorbidities or age to 5 ME/CFS onset types. Thirty-four comorbidities were considered in a logistic regression model of the form OnsetType ~ age + sex + comorbidities and an intercept. A covariate is only shown if it survived Bonferroni multiple testing correction ( *p*<0.05) for one or more onset type. Active and inactive comorbidities were considered independently: Active, if the condition has given symptoms in the past 6 months, or Inactive, if the condition has not given symptoms in the past 6 months, either because it has died down or treatment has controlled it. The z-score (Y-axis) is the effect-size estimate in standard deviation units.

### ME/CFS after glandular fever mostly affects adults a decade after peak incidence

We first analysed participants’ ages at ME/CFS onset. Onsets occurring >5y ago do not allow fine resolution of their dates, especially for those responding “Between 5 and 10 years” or “Over 10 years” to “How long have you had your illness?” Consequently, we only considered participants reporting onsets within the last 5y ( *n*=3,150). The median age of this group was 40y, IQR=31y-51y, implying that most participants’ onsets occurred between 25y and 50y of age. This is older than the peak incidence of glandular fever in the UK (15y-19y old) ^15^. Rather than most participants reporting ME/CFS onset in the last 5y after glandular fever being in their early twenties, as expected, they were a decade older (median ages 30.5y [IQR=23y-41y]). This difference is consistent with adolescents being less likely, than older people, to develop ME/CFS after glandular fever.

### ME/CFS comorbidities and symptoms are sex- and age-biased

The substantial number of males participating in DecodeME ( *n*=2,827) allowed the study to reveal previously unreported sex-biases in comorbidities or symptoms. Females with ME/CFS reported more comorbidities and symptoms than males in the DecodeME questionnaire. Two-thirds (66.7%; *n*= 9,507) of females, but a half (52.7%; *n*=1,489) of males, reported at least one active comorbidity; similarly 39.2% ( *n*=5,588) of females and 28.6% ( *n*=809) of males reported at least one inactive comorbidity. Female participants reported, on average, more symptoms than males (42 versus 36).

To test more formally for an association between age and sex and each symptom we used logistic regression and a Bonferroni correction to adjust for multiple testing (Methods). This identified 62 of 80 symptoms as significantly female-biased, and 61 as biased towards younger age ( Figure 4). Female-bias is evident across all symptom types ( Figure 4). Females were significantly more likely to report fatigue ‘often, repeatedly, or all the time’ ( *p=*5.9×10 ^-4^; age *p*=1.1×10 ^-13^), and more likely to report post-exertional malaise after physical or mental activity ( *p*=2.8×10 ^-4^; age *p*=4.2×10 ^-7^).

The type of infectious or non-infectious disease onset does not explain these strong and pervasive sex-biases because proportions of females were not significantly different across the five onset types (83.1%-84.5%; χ ^2^ = 1.707, df = 4, *p* = 0.79).

### Factors, comorbidities and symptoms associated with ME/CFS severity

Being female, increasing age and being over 10y from ME/CFS onset are each separately associated with severity in the DecodeME cohort (sex: *p*=4.5×10 ^-4^; age: *p*<2.2×10 ^-16^; years since ME/CFS onset: *p*=1.6×10 ^-6^). These results are from a comparison of those with mild ME/CFS (34%; *n*=5,779) against the remaining 66% ( *n*=11,295) with moderate, severe or very severe illness. Testing for all 68 co-occurring (active and inactive) comorbidities, and including both age and sex as covariates in the model, 6 active comorbidities were significantly associated with severity. In order of decreasing significance these were: fibromyalgia ( *p*<2×10 ^-16^), clinical depression ( *p*<2×10 ^-16^), irritable bowel syndrome ( *p*=5.7×10 ^-12^), mast cell activation syndrome ( *p*=1.8×10 ^-11^), diabetes ( *p*=9.5×10 ^-10^) and sleep apnoea ( *p*=5.2×10 ^-8^). Severity was also associated with a single inactive comorbidity, hypothyroidism ( *p*=1.6×10 ^-5^).

Testing all symptoms simultaneously with sex and age, showed strong association between ME/CFS severity and 18 factors including fatigue, age, difficulty remaining standing, and sleep problems ( Figure 5). Finally, participants describing their illness as relapsing and remitting were significantly less likely to report their illness as moderate, severe or very severe than those reporting fluctuating symptoms ( *p*<2.2×10 ^-16^).

### Length of illness, symptoms and comorbidities differ by onset type

A feature that strongly distinguished among the five onset types was longevity of participants’ ME/CFS symptoms. Participants reporting an infection at onset were more likely to have had ME/CFS symptoms for over 10y than those reporting no infection at onset (66.8% [ *n*=7,246] vs. 45.1% [ *n*=1,183]). This is despite their similar ages (medians 54y [IQR=43y-64y] and 52y [IQR=41y-62y], respectively).

The statistical significance of this difference is strong. When testing for association between those with an infection around the time of ME/CFS onset and duration (<10y vs. >10 years since time of onset), age and sex, only association with duration was significant ( *p* = 4×10 ^-67^). This relative paucity of participants not reporting an infection around the time of onset of their ME/CFS over 10y ago is unexpected, and not easily explained by historic variation in ME/CFS triggers because association with age was not significant in this analysis ( *p* > 0.05). When analysed separately, each onset type was not associated with participants’ sex at birth, when including age and ME/CFS duration over 10y in the analysis.

Significant differences occurred between the 5 ME/CFS onset types and 4 fatigue symptoms ( Figure 6A), 16 other symptoms ( Figure 6B) and 3 different types of illness course ( Figure 6C). Those with glandular fever onset were significantly more likely than others to report swollen or tender glands and viral infections with long recovery periods within the last 6 months, and to experience relapsing and remitting symptoms (relative to ‘Fluctuating’, the majority response). Others with COVID-19 infection at ME/CFS onset preferentially reported a tight feeling in the chest, sensitivity to alcohol and a feeling of burning in the lungs. Participants with other types of infection onset more frequently reported feeling mentally fatigued, feeling fatigued less than half the time, and difficulties remaining standing, and less frequently reported feeling more sleepy than is normal, having worsening symptoms (relative to ‘Fluctuating’), unusual changes in appetite and mood swings.

Participants reporting an infectious onset (when compared to those who did not) were also significantly more likely to report: improving symptoms, relapsing/remitting, or recovered (relative to ‘Fluctuating’) symptoms, and less likely to report worsening symptoms (again, relative to ‘Fluctuating’). They were more likely, among other things, to report viral infections with long recovery periods, fewer viral infections than they used to get, and having a pale face. Other symptoms that were significantly more likely to be reported by participants without an identified infection at onset were fatigue more than half the time, reduced libido, and unusual changes in appetite. They were also less likely to report symptoms common during infection: flu-like feelings, and swollen or tender glands.

Those with an infection around the time of onset of ME/CFS more frequently reported symptoms typical of infection in the last 6 months, whereas those reporting no infection at onset less frequently indicated these symptoms. This was unexpected because of the long time-lag between onset (mostly >10y ago) and participants’ recent questionnaire responses. Even though most participants report a long interval between their onset of ME/CFS (mostly >10y ago) and their recent symptoms characteristic of infection, our results cannot distinguish between whether these recent symptoms are a natural consequence of their ME/CFS onset, for example because of viral persistence in some individuals ^17^, or else they are independent of onset.

In our final analysis, we tested for association between participants’ onset type and their comorbidities, age and sex. Only younger age, rather than any comorbidity, was significantly associated with glandular fever onset ( Figure 7). Among all onset types, only coronavirus disease 2019 (COVID-19 caused by SARS-CoV-2) infection was significantly associated active Mast Cell Activation Syndrome (MCAS), i.e. MCAS symptoms within the previous 6 months. COVID-19 related onset was also negatively associated with active fibromyalgia. Onset with another infection was positively associated with inactive Shingles or active Lyme disease, and negatively associated with fibromyalgia or clinical depression. Onset without reported infection at onset was significantly associated with recent clinical depression symptoms; and, onset with unknown infection status was significantly associated with active fibromyalgia as a comorbidity ( Figure 7).

In summary, we report significant associations to five onset types derived from participants’ responses to the question ‘Did you have an infection when, or just before, your first ME/CFS symptoms started?’:

1. ‘ **Yes, glandular fever**’ (17%; *n*=2,936): These participants were more likely to report swollen or tender glands and viral infections with long recovery periods, and to experience relapsing and remitting symptoms.
2. ‘ **Yes, COVID-19**’ (2%; *n*=380): These participants were more likely to report having Mast Cell Activation Syndrome, a tight feeling in the chest or a burning feeling in the lungs. Mast cell activation symptoms are prevalent in Long-COVID ^18^ but this condition is rarely diagnosed in people with ME/CFS ^19^ although perhaps because only recently have MCAS diagnostic criteria been defined ^20^.
3. ‘ **Yes, another infection**’ (44%; *n*=7,537): These participants were more likely to be mentally fatigued, to report viral infections needing long recovery periods, and to have had Shingles in the past or symptomatic Lyme disease in the last 6 months. They were also less likely than others to report active clinical depression or fibromyalgia. Over 100 types of infections have been reported to occur at ME/CFS onset ^11^.
4. ‘ **No**’ (i.e. no infection at onset; 16%; *n*=2,625): These participants were more likely to report fatigue more than half of the time, to feel nauseous, and to have recent clinical depression symptoms.
5. ‘ **Don’t know**’ (21%; *n*=3,596): These were more likely to report fibromyalgia as a comorbidity, and less likely to report cold or flu-like symptoms, improving or relapsing and remitting symptoms.

## Discussion

DecodeME questionnaire responses from *n*=17,074 participants reveal how people, who report being diagnosed with ME/CFS, do not form a single homogeneous group. Although this was long suspected ^21^, it had not previously been substantiated using a large country-wide cohort ascertained using a single protocol. More specifically, the cohort’s heterogeneity was most evident in four respects: (1) large and statistically significant differences among five ME/CFS onset types, relating to their different associations to symptoms, comorbidities and illness severity; (2) the greater likelihood of participants to have longstanding (>10y) ME/CFS symptoms if they report an infection at onset; (3) substantial differences between females and males in their symptoms and comorbidities; and (4) greater disease severity for those who are female, older and/or have had ME/CFS for >10y.

This initial DecodeME cohort has a comparable age-distribution to previous USA-based studies ^22– 24^, the reported comorbidities ( Figure 2) are similar to those of a previous study ^25^, and proportions of participants reporting glandular fever or another infectious disease around the time of onset are similar to those previously reported ^11, 25, 26^. The DecodeME cohort’s females outnumber males by over five-to-one, which is one of the highest female-bias among those with ME/CFS yet reported internationally ^3, 9, 27– 32^. Cohorts of these previous questionnaire studies numbered in the hundreds. DecodeME’s larger cohort thus provides robust statistical support to these previous findings from less well-powered studies.

Studies involving hundreds of participants previously concluded that ME/CFS exhibits few sex differences in illness patterns ^33, 34^. Smaller studies indicated older age as associated with greater ME/CFS symptom severity, but other studies found no such association (reviewed in 12, 35). These previously limited cohort sizes did not permit comprehensive analysis. In a previous study, three symptoms were reported significantly more often by females than males: fever, swollen glands, and sore throat ^34^. In our study, we replicated these findings, and found a further 59 of 80 ME/CFS symptoms that are also female-biased. Our analyses additionally found 61 symptoms biased towards younger age, with only 5 biased towards older age.

The raw number of symptoms may not be meaningful, however, as symptoms can be overlapping, and people with ME/CFS may, over time, pace sufficiently to avoid triggering some symptoms or may begin to describe their symptoms with fewer labels, particularly when interventions are not available to treat each symptom effectively. Indeed, rather than younger participants reporting increased severity, we found that being female, older and over 10y from onset are all risk factors for ME/CFS severity.

The median time to receive a clinical diagnosis in the UK is 2 years which is reflected in DecodeME participants’ responses. Specifically, participants whose illness started within the last 1-3y or 0.5-1y ( *n* = 1,287 and 177) were respectively 21% and 57% fewer per year than the study’s participants from the 3-5y recruitment interval ( *n*=1,634). 

Despite its large cohort size (N=17,074), extensive community reach and use of paper, as well as online, questionnaires, the analysis presented here – of the December 2022 DecodeME data freeze – has four main limitations. First, recruitment is restricted to participants over the age of 16y, which limited investigation of paediatric ME/CFS. Second, when asking participants if they were diagnosed by a health professional we did not require clinical confirmation of reported answers. Nevertheless, our extensive engagement with participants and the internal consistency of their responses encourage us to believe that questionnaire answers have been given in good faith, noting that inconsistent responses may result from respondents’ ME/CFS symptoms including their cognitive dysfunction. Third, most ME/CFS symptoms are not independent of one another. Consequently, multicollinearity should be borne in mind for those analyses considering multiple symptoms in the same analysis. Fourth, regrettably DecodeME has not yet been successful in recruiting proportionately from minoritised groups. There is little consensus on whether ME/CFS prevalence differs among these and other groups ^36^. Other recruitment and representativeness biases are also possible, as with all research cohorts.

A previous study indicated that ME/CFS onset type associates with severity ^37^ although this was not replicated by our larger study. Instead, we identified large numbers of comorbidities and symptoms that are each more likely to be reported by participants with a specific onset type.

These onset types reveal differences amongst those with ME/CFS regarding their symptoms and comorbidities ( Figure 4). However, these distinctions are not absolute. For example, those reporting no infection at onset (Type 4, above) are not cleanly distinguished from all others by active clinical depression. Rather, they were the only onset type that was more likely to report this diagnosis (25.4%; *n*=667) than all other participants were (19.6%; *n*=2829). Similarly, Type 3 (“other infection”) contains a higher proportion (9.4%; *n*=710) of those who report inactive shingles, than all other participants (7.3%; *n*=692). Shingles is caused by reactivation of latent varicella-zoster virus (a herpesvirus). People with herpes zoster infection are known to have a significantly higher risk of ME/CFS up to at least 6 years ^38^ fuelling speculation that varicella-zoster virus infection is a cause of ME/CFS that may be prevented by vaccination. 2.5% of ME/CFS cases have been attributed to varicella-zoster virus infection ^11^. We note that among those reporting no infection around the time of onset (Type 4) some may have developed ME/CFS secondary to an infection without an obvious acute phase, such as can occur with Epstein-Barr virus ^39^. However, we are unable to test this hypothesis here.

ME/CFS’ poor long-term prognosis, its severe symptoms – especially for older females, its profound impact on the quality of life of people with ME/CFS and their family members ^9, 32^, and its high population prevalence (>0.2%) ^1^ present formidable healthcare and research challenges. Considering that 64% ( *n*=10,853) of DecodeME participants reported an infection around the time of onset, any vaccination against the major infectious agents triggering ME/CFS, including Epstein-Barr virus ^40^, SARS-CoV-2 ^41^ and influenza viruses ^42^ may help reduce ME/CFS incidence in the future, especially for individuals more susceptible to severe disease, or those more likely to be exposed to the infectious agents.

Formal investigation of each onset type’s significance is now warranted. In order to address effectively the devastating impact that ME/CFS has on the millions of people worldwide affected, the research community and policy-makers will need to give sustained focus on disease classification and aetiology. It is for this reason that DecodeME is seeking to identify genetic factors causal of altered ME/CFS risk ^14^ and will do so for infectious versus non-infectious onset participants separately and combined, if final recruitment numbers allow. Recruitment to the DecodeME study is ongoing. Results will be updated after the project’s recruitment phase has ended.

## Data Availability

Extended Data is available from the Open Science Framework (OSF) website: https://osf.io/rgqs3/. This Project site contains CC-BY license DecodeME Study Documents: (i) the DecodeME Questionnaire (version 6) annotated by question identifier (Qid) which were used in the logistic regression analyses detailed in (ii) the regressionResults.txt file. DecodeME anonymised data allowing investigation of this study’s consented data are available to researchers by managed access via a Data Access Committee, https://www.decodeme.org.uk/faqs/who-will-be-able-to-use-my-data-and-sample/. This committee consists of a scientist, a patient and a charity representative who strictly control access to the data. DecodeME’s anonymised and consented data are only shared with studies that meet high standards and whose academic or industrial researchers agree to treat its data with respect and to keep it secure.
