# Supplementary material for: Typing myalgic encephalomyelitis by infection at onset: A DecodeME study
Source: NIHR Open Res. 2023 Aug 21;3:20. Originally published 2023 Apr 24. [Version 4] doi: 10.3310/nihropenres.13421.4 (PMC10593357; doi:10.3310/nihropenres.13421.4)
Supplement: Peer review history

# Reviewer report: Reviewer response for version 4

**DOI:** 10.3310/nihropenres.14615.r30248

**Published:** 2023-08-22

**Authors:** Sarah Tyson

**Affiliations:** 1 Division of Nursing, Midwifery & Social Work, School of Health Sciences, Faculty of Biology, Medicine and Health, The University of Manchester, Manchester, England, UK

I am happy with the revisions the authors have made

Is the work clearly and accurately presented and does it cite the current literature?

Partly

If applicable, is the statistical analysis and its interpretation appropriate?

I cannot comment. A qualified statistician is required.

Are all the source data underlying the results available to ensure full reproducibility?

Yes

Is the study design appropriate and is the work technically sound?

Partly

Are the conclusions drawn adequately supported by the results?

Partly

Are sufficient details of methods and analysis provided to allow replication by others?

No

Reviewer Expertise:

pwME, clinical researcher academic

I confirm that I have read this submission and believe that I have an appropriate level of expertise to confirm that it is of an acceptable scientific standard.

# Reviewer report: Reviewer response for version 3

**DOI:** 10.3310/nihropenres.14604.r29994

**Published:** 2023-08-14

**Authors:** Sarah Tyson

**Affiliations:** 1 Division of Nursing, Midwifery & Social Work, School of Health Sciences, Faculty of Biology, Medicine and Health, The University of Manchester, Manchester, England, UK

This paper is progressing well and I appreciate the addition of the final paragraph of the discussion. However, it is premature to suggest that *"Therefore, in future research studies, subcategorising ME/CFS by onset type may help to reduce cohort heterogeneity and aid scientific discovery..."*

 Undoubtedly finding an effective way to categorise ME/CFS would be a big step forward, but the data presented in this paper do not support onset type for this use, at present. 

 The data indicate that onset groups show some differences in presentation, and many similarities. Further research is needed to establish whether onset type is a useful way to categorise pwME/CFS and to compare it to other methods, such as disease severity or symptom clusters before it can recommended for use in clinical practice or research.

 It is fine to raise the potential benefits of an effective way to categorise ME/CFS, but greater caution is needed in suggesting onset type as the way of doing so.

Is the work clearly and accurately presented and does it cite the current literature?

Partly

If applicable, is the statistical analysis and its interpretation appropriate?

I cannot comment. A qualified statistician is required.

Are all the source data underlying the results available to ensure full reproducibility?

Yes

Is the study design appropriate and is the work technically sound?

Partly

Are the conclusions drawn adequately supported by the results?

Partly

Are sufficient details of methods and analysis provided to allow replication by others?

No

Reviewer Expertise:

pwME, clinical researcher academic

I confirm that I have read this submission and believe that I have an appropriate level of expertise to confirm that it is of an acceptable scientific standard, however I have significant reservations, as outlined above.

# Reviewer report: Reviewer response for version 2

**DOI:** 10.3310/nihropenres.14591.r29746

**Published:** 2023-07-13

**Authors:** Sarah Tyson

**Affiliations:** 1 Division of Nursing, Midwifery & Social Work, School of Health Sciences, Faculty of Biology, Medicine and Health, The University of Manchester, Manchester, England, UK

Thank you for asking me to review this resubmission. The authors have obviously undertaken a lot of work and the paper is much improved. Congratulations. It is now much easier to follow and the scientific merit is clear. I am happy to accept the changes made, except I would like the authors to reconsider one point, which is now starker given the helpful revisions in the paper. 

 I am not really clear whether the authors have answered the original research question – are there different types of ME/CFS? Are they saying –yes, illness onset defines different types of ME? There seems to be some leaning towards this interpretation but it isn’t stated explicitly. If so, I am not convinced. Not because of anything in the method or analysis (as far as I can tell, that is fine), but because they started with the assumption that onset type (and they predefined the types of onset) was the definitive defining variable. I’m no statistician, but I think in a cohort and dataset of this size, one is bound to find some associations and significant differences. The issue is the importance of those found. 

 The essential section of the discussion regarding the importance of these findings and their potential application in future research and clinical practice is missing, possibly because the authors aren’t sure themselves.

 I think this comes down to the founding basis of the paper; that it started with illness type as the defining characteristic, and had pre-defined the types of onset. What was the rationale for this choice? There is a logic to it, but would other characteristics provide clearer differentiation, or be more relevant to future research efforts or clinical applications? Viral/non-viral onset; severity of symptoms or disability; outcome/course of illness (in terms of recovery, fluctuations deterioration etc); or symptomology according to the body system(s) affected are also obviously clinically applicable and meaningful. I’m not saying that illness onset isn’t a (or maybe THE) way to categorise ME, but that to be adopted in practice or research it needs to show a clearer differentiation between types than other possible dependent/ defining variables. I.e. it needs to be the best way of typing PwME, not just a possible way of doing so.

 From what I can gather, the upshot of a section on the importance and application of the findings might be something along the lines of …. *“The findings show that sex and age, although associated with symptomology, co-morbidities and disease severity, are not factors that define different types of ME. Type of onset does show some significant differences in terms of symptomology, co-morbidity and illness severity, however these differences are not absolute and using ‘type of onset’ to define different types of ME would be premature. Further research is needed to investigate the accuracy with which ‘type of onset’ (probably with additional variables) can categorise pwME, and also to explore other potential ways defining variables, before comparing these with onset type to establish the most effective way to categorise type of ME.”*

Is the work clearly and accurately presented and does it cite the current literature?

Partly

If applicable, is the statistical analysis and its interpretation appropriate?

I cannot comment. A qualified statistician is required.

Are all the source data underlying the results available to ensure full reproducibility?

Yes

Is the study design appropriate and is the work technically sound?

Partly

Are the conclusions drawn adequately supported by the results?

Partly

Are sufficient details of methods and analysis provided to allow replication by others?

No

Reviewer Expertise:

pwME, clinical researcher academic

I confirm that I have read this submission and believe that I have an appropriate level of expertise to confirm that it is of an acceptable scientific standard, however I have significant reservations, as outlined above.

# Reviewer report: Reviewer response for version 2

**DOI:** 10.3310/nihropenres.14591.r29745

**Published:** 2023-07-13

**Authors:** Suzanne Vernon

**Affiliations:** 1 The Bateman Horne Center of Excellence, Salt Lake City, UT, USA

Thank you for making the revisions to this interesting manuscript.

Is the work clearly and accurately presented and does it cite the current literature?

Partly

If applicable, is the statistical analysis and its interpretation appropriate?

Yes

Are all the source data underlying the results available to ensure full reproducibility?

Yes

Is the study design appropriate and is the work technically sound?

Partly

Are the conclusions drawn adequately supported by the results?

Partly

Are sufficient details of methods and analysis provided to allow replication by others?

Partly

Reviewer Expertise:

Clinical research of ME/CFS and PASC

I confirm that I have read this submission and believe that I have an appropriate level of expertise to confirm that it is of an acceptable scientific standard.

# Reviewer report: Reviewer response for version 1

**DOI:** 10.3310/nihropenres.14555.r29219

**Published:** 2023-05-25

**Authors:** Suzanne Vernon

**Affiliations:** 1 The Bateman Horne Center of Excellence, Salt Lake City, UT, USA

This is a study uses responses to the DecodeME questionnaire to identify ME/CFS subtypes based on self reported type of infection onset that coincided with ME/CFS symptom onset. The choices included "Yes, glandular fever", "Yes, COVID-19", "Yes, another infection", "No", or "Don't know". Responses to this question forms the basis for the symptom analysis and subtyping. 

 The authors claim that more comorbidity's in females have not been previously reported. However, there are studies by Jason et al ^1^ and Jones et al ^2^ that also reported this. Including these important citations would further strengthen the findings of this paper. A couple of citations are provided for your consideration. There are many more that would support these results.

 Involvement of patients and the public in the study design is important and adds essential lived experience(s) to the overall DecodeME study design. (This section of the methods should be written more succinctly). However, it is not clear why a standardized and well-validated questionnaire(s), for example the DePaul Symptom Questionnaire (DSQ) was not used rather than or in addition to the DecodeME Questionnaire. The DSQ is validated for ME/CFS, has been used worldwide, and is available in a number of languages. It is also used in the collection of core data elements for ME/CFS (as developed by NIH/NINDS). The use of a new questionnaire that has not been validated for ME/CFS limits the generalizability of these results. Finally, the inclusion of people with comorbid diseases that are exclusionary could explain symptoms limits the ability to replicate these results.

 How does a paper questionnaire help decrease or eliminate bias?

 The results describe 80+ symptoms yet the DecodeME Questionnaire asks about 12 major symptoms. What accounts for this discrepancy. If each item listed under each major symptom was considered as a separate system, how was collinearity accounted for?

 What was the positive test for those that responded "Yes, other infection"?

 Duplicated text in the results, 2nd to last paragraph in Results.

 The authors point out in the discussion that previous work associated onset type with disease severity and that this study did not find this. Could this be due to the lack of clear severity and frequency assessment of symptoms in the DecodeME Questionnaire?

 The major strength of this paper is the >17,000 respondents.

 The major weaknesses of this paper are the use of a new ME/CFS questionnaire and the inclusion of exclusionary diseases that could explain the reported symptoms.

Is the work clearly and accurately presented and does it cite the current literature?

Partly

If applicable, is the statistical analysis and its interpretation appropriate?

Yes

Are all the source data underlying the results available to ensure full reproducibility?

Yes

Is the study design appropriate and is the work technically sound?

Partly

Are the conclusions drawn adequately supported by the results?

Partly

Are sufficient details of methods and analysis provided to allow replication by others?

Partly

Reviewer Expertise:

Clinical research of ME/CFS and PASC

I confirm that I have read this submission and believe that I have an appropriate level of expertise to confirm that it is of an acceptable scientific standard, however I have significant reservations, as outlined above.

## References

[1] : Variability in diagnostic criteria for chronic fatigue syndrome may result in substantial differences in patterns of symptoms and disability. *Eval Health Prof* .2003;26(1) : 10.1177/0163278702250071 3-22 10.1177/0163278702250071 12629919

[2] : An evaluation of exclusionary medical/psychiatric conditions in the definition of chronic fatigue syndrome. *BMC Med* .2009;7: 10.1186/1741-7015-7-57 57 10.1186/1741-7015-7-57 19818157PMC2768736

# Reviewer report: Reviewer response for version 1

**DOI:** 10.3310/nihropenres.14555.r29220

**Published:** 2023-05-18

**Authors:** Sarah Tyson

**Affiliations:** 1 Division of Nursing, Midwifery & Social Work, School of Health Sciences, Faculty of Biology, Medicine and Health, The University of Manchester, Manchester, England, UK

The results of the DeCode have been eagerly awaited, even the interim results so there is bound to be a lot of interest in this paper. However I feel it needs some more work to make it as easy to understand as possible. The lack of clarity stems primarily (I think) from a uncertainty about the aim/objective/research questions addressed in this paper. It has a feel of something of a fishing exercise, and having gotten some results, the authors are keen to tell people about them (which is admirable) but aren’t really clear about what and why. The methods section, in particular needs more details so the reader can understand what was done, why and how.

 To look at each detail in turn:

**Abstract **

- The abstract needs a clear aim/objective/res question and further methodological detail. At present there is no info about the questionnaire, except that there was one. An indication of the selection criteria, recruitment methods, and details of the questionnaire (what does it measure, how?) And the analysis methods (which need to be linked research questions) are needed. In the results section there needs to be some data and the unique findings made clearer. It is hardly news that ME/CFS is a heterogeneous condition.
- The plain English summary includes rather lot of jargon. For example – “For many people, ME/CFS means a substantial impairment of their activity levels, a high level of disability and a poor quality of life” rather than *“for many people, ME/CFS means disability and poor quality of life ..”* NB. The PSP included a range of HCPs not just Doctors,

**Methods**

- I appreciate that PPI has been central to the DeCode project but there needs to be further details of the actual methods used. The 3 ^rd^ paragraph of the methods isn’t really relevant to what was done in this project and can be removed.
- The description of the questionnaire needs to include its aim; an outline of the content (what does it ask about?), its size/length, types of questions and response format(s). The details of how it was developed and validated and any other psychosomatics would be useful. The reader needs to know what data were collected and how, and be reassured that the information obtained was relevant and accurate ie psychometrically robust.
- The paragraph on the ‘cohort’ describes the process of completing part of the questionnaire. It would be easier to understand who was recruited to the study and how by listing the selection criteria, and stating the recruitment strategies- where and how were people recruited? I think it was largely via social media and support groups which is fine. Just say so.
- The paragraph on significance testing needs to be replaced by an explanation of the analysis used, linked to the objectives/ research questions. It might be helpful to get a statistician to help with this. I see that logistic regression model(s) was/were used. This needs further detail, particularly the dependent and independent variables. There is reference to figures which are not explained and I cannot really follow. Also Onset Type, symptoms (in groups with different numbers), severity, illness courses, ‘Fluctuating’, the majority response, and comorbidities are unexplained, undefined and unconnected to any objective or research question. This section is key to understanding the whole paper so needs to clearly describe what was analysed, why and how.

**Results**

- The 1 ^st^ paragraph (after the 1 ^st^ sentence) describes what participants did rather than what was found, and so belongs in the method section. The results need to start with a summary description of the sample – the number, mean age; sex ratio; time since diagnosis +/or duration of symptoms. Then the main co-morbidities; frequency of symptoms etc. The text needs to be understandable without the tables/figures and vice versa. The figures /tables add extra detail but there needs to summary in the text.
- The results section needs to only include what was found in this study. Reflections on how they relate to other studies (or not) are for the discussion (see paragraph 2).
- In the summary of results, try to avoid copy and pasting the results as they were churned out. Eg rather than “Participants were asked: “Did you have an infection when, or just before, your first ME/CFS symptoms started?” with five possible responses: (i) Yes, glandular fever ( *n*=2,936), (ii) Yes, COVID-19 ( *n*=380), (iii) Yes, another infection ( *n*=7,537), (iv) No ( *n*=2,625), or (v) Don’t know ( *n*=3,596; Figure 3). Proportions of people in the first 3 categories reporting a positive laboratory test of their infection prior to ME/CFS were 68.4%, 50.5% and 25.9%, respectively“ – which is lengthy and unfocussed Summarise this for the reader by saying (for example) “ *Most (n, %) reported an infectious onset to their symptoms, most commonly glandular fever (17%, n=xx) this was glandular fever, followed by covid-19 (n,%). However only 68% and 51% respectively of respondents with these triggers reported a positive laboratory test confirming the diagnosis”*.
- Do not include reference/comparison to other studies in the results section; this is for the discussion section.
- Several of the sentences are rather convoluted and a bit more plain English would not go amiss. For example, I eventually managed to work out that “Participants who started their illness within the last 1–3y or 0.5–1y numbered 1,287 or 354, respectively. These numbers are 57% and 21% fewer, per year, than the study’s 1,634 participants from the 3–5y recruitment interval.” Actually meant *“Most *1,634 *(xx%) were diagnosed 3-5 years previously, followed by 1287 (x%) with 1-3 year duration and 354 (X%) were more recently diagnosed (0.5-1 year)”.*
- Always include both the number and % of participants being referred to.
- Avoid any interpretation in the results.
- Whenever presenting an average value an indication of the variability (whether standard deviations for means, or inter-quartile ranges for medians) is needed.
- Why was a sub-group used to assess incidence? I don’t think ‘incidence’ is the proper term. The reference to the frequency with which ME/CFS occurs within a defined general population and this dataset doe sot include that data.
- My comments above also apply to the rest of the results. At present they are difficult to read and to work out the main message from the rests. The reader is left to work out the main gist of the results themselves and it is far from easy.

**Discussion**

- The 1 ^st^ paragraph of the discussion needs to summarise the main findings; the headline news in sufficient detail to the reader to have an insight into the significance of the results. Merely saying ME/CFS is heterogeneous with substantial differences in onset; symptoms, comorbidities and severity and sex differences isn’t new, we already know this. The headline news needs some (summary) details about what the differences were.
- Then you compare your findings with previous studies – which is great. Make sure all the comparisons and interpretation are here and not in the results. Also try to include any possible explanation for differing/contrasting results (usually due to sample size, method of data collection or selection criteria)
- The limitations section is good.
- The list of symptoms and other characteristics associated with different onset types are new data and belong in the results section – and would help a great deal to understand the results. Although I am a bit confused - why/how was onset type used to sub-group pwME? why not look at cluster/co-occurrence of symptoms and/or co-morbidities +/- onset? It may be that this was done and onset was the strongest factor, if so it needs to be explained more clearly.
- What is the clinical or significance/ implications of the findings? Are you saying onset is such an important/explanatory factor that it should be used to group different types of ME/CFS? Or that they seem the best bet (if so, I didn’t pick that up) or what?
- I'm presuming there isn’t a conclusion section where the authors relate the findings to the original objectives /research questions and whether they have been fulfilled, because the journal specifically excludes one. If not, please add a conclusion.

Is the work clearly and accurately presented and does it cite the current literature?

Partly

If applicable, is the statistical analysis and its interpretation appropriate?

I cannot comment. A qualified statistician is required.

Are all the source data underlying the results available to ensure full reproducibility?

Yes

Is the study design appropriate and is the work technically sound?

Partly

Are the conclusions drawn adequately supported by the results?

Partly

Are sufficient details of methods and analysis provided to allow replication by others?

No

Reviewer Expertise:

pwME, clinical researcher academic

I confirm that I have read this submission and believe that I have an appropriate level of expertise to state that I do not consider it to be of an acceptable scientific standard, for reasons outlined above.
